# Genetic and Non-Genetic Mechanisms Underlying Cancer Evolution

**DOI:** 10.3390/cancers13061380

**Published:** 2021-03-18

**Authors:** Yelyzaveta Shlyakhtina, Katherine L. Moran, Maximiliano M. Portal

**Affiliations:** Cell Plasticity and Epigenetics Lab. Cancer Research UK—Manchester Institute, The University of Manchester, Alderley Park, SK10 4TG, UK; lisa.shlyakhtina@cruk.manchester.ac.uk (Y.S.); Katherine.moran@cruk.manchester.ac.uk (K.L.M.)

**Keywords:** cancer evolution, genetic heterogeneity, non-genetic heterogeneity, plasticity, tumour

## Abstract

**Simple Summary:**

Our manuscript summarizes the up-to-date data on the complex and dynamic nature of adaptation mechanisms and evolutionary processes taking place during cancer initiation, development and progression. Although for decades cancer has been viewed as a process governed by genetic mechanisms, it is becoming more and more clear that non-genetic mechanisms may play an equally important role in cancer evolution. In this review, we bring together these fundamental concepts and discuss how those tightly interconnected mechanisms lead to the establishment of highly adaptive quickly evolving cancers. Furthermore, we argue that in depth understanding of cancer progression from the evolutionary perspective may allow the prediction and direction of the evolutionary path of cancer populations towards drug sensitive phenotypes and thus facilitate the development of more effective anti-cancer approaches.

**Abstract:**

Cancer development can be defined as a process of cellular and tissular microevolution ultimately leading to malignancy. Strikingly, though this concept has prevailed in the field for more than a century, the precise mechanisms underlying evolutionary processes occurring within tumours remain largely uncharacterized and rather cryptic. Nevertheless, although our current knowledge is fragmentary, data collected to date suggest that most tumours display features compatible with a diverse array of evolutionary paths, suggesting that most of the existing macro-evolutionary models find their avatar in cancer biology. Herein, we discuss an up-to-date view of the fundamental genetic and non-genetic mechanisms underlying tumour evolution with the aim of concurring into an integrated view of the evolutionary forces at play throughout the emergence and progression of the disease and into the acquisition of resistance to diverse therapeutic paradigms. Our ultimate goal is to delve into the intricacies of genetic and non-genetic networks underlying tumour evolution to build a framework where both core concepts are considered non-negligible and equally fundamental.

## 1. Introduction: Intra-Tumour Heterogeneity Infers Tumour Evolution

Since the early 1950s, the observation that most neoplasms display significant genetic heterogeneity has gained acceptance in the scientific community. More precisely, it was during the year 1957 that Julian Huxley published a landmark perspective on cancer, summarizing that solid tumours are not genetically homogeneous, but contain cells that display different ploidy and structural chromosomal rearrangements [1]. Moreover, he conceptualized at the time that it was quite probable that not all aberrations will lead to a viable phenotype in the context of tumours. It was only two decades later that Peter Nowell formalized a model proposing the principles governing the evolution of cancer cell populations [2]. According to his model, tumours originate from a single “normal” cell that acquires a mutation, which provides a growth advantage over normal cells within its niche, thus leading to clonal expansion. Nowell insightfully recognized that tumour cells may be characterized by genetic instability and proposed that over time new mutations are accumulated in a stepwise manner, therefore generating sub-clonal populations; however, due to natural selection, most of the mutant sub-populations are eliminated and only one of them becomes the predominant sub-clone. Notably, Nowell’s model strongly recapitulates the concepts laid down in linear Darwinian evolution, where it is suggested that reproductive individuals, in this case tumour cells, acquire genetic alterations in a purposeless manner followed by the natural selection of the fittest variant. In the following decades, a wealth of experimental evidence has been generated which supports this line of thought, showing that throughout the development of a tumour, cancer cells are indeed subjected to a diverse array of selective pressures that may shape their evolutionary trajectory, firstly within their niche of origin and further upon the invasion of distant tissues.

Importantly, with the advent of next-generation sequencing (NGS) technologies, large-scale genomic studies have demonstrated that the evolution of cancer cell populations may be much more complex than originally thought and may not simply follow classic linear Darwinian evolution. In that regard, after analysing thousands of human tumours of different origins, it has been found that the number of mutations that can be detected within primary tumours, or are found to be heterogeneously represented between primary and metastatic lesions, vary from a handful to thousands [3,4,5,6,7,8,9,10,11,12,13,14,15,16]. These data argue that tumours may contain more than a single predominant clone and suggest that intra-tumour heterogeneity arises as a consequence of the simultaneous coexistence of multiple sub-clonal populations. In recent years, the development of single-cell DNA sequencing (scDNA-seq) technologies has provided a high-resolution view on the complex sub-clonal architecture of tumours and allowed the reconstruction of lineage trees depicting branched clonal evolution in cancer, thus validating cancer development as an evolutionary process [17,18,19,20,21,22,23]. Importantly, it has become increasingly evident that, besides genetic heterogeneity, tumours may also display significant non-genetic heterogeneity often referred to as phenotypic variability [24,25,26,27,28,29,30,31,32]. Notably, phenotypically distinct but genetically identical sub-clones may display stable states that exhibit drastic variations in their responses to environmental cues [24,27,33,34]. Thus, it is reasonable to hypothesise that both genetic and non-genetic heterogeneity may play a pivotal role in shaping the evolutionary trajectory in cancer cell populations, a crucial point in this review that will be further discussed in the following sections.

## 2. Challenges in Understanding Cancer Evolution

Despite the fact that a significant progress in assessing intra-tumour heterogeneity (ITH) has been achieved, it remains largely elusive how and at which stage of tumour development ITH emerges, how it is maintained during tumour development and progression and whether there is a functional significance to such heterogeneity. This apparent vacuum in knowledge stems directly from the lack of adequate technologies to trace and explore the tumour evolutionary landscape at the single cell level in vivo. For example, it has been long acknowledged that the genetic mechanisms underlying cancer initiation and progression do not only consist of point mutations in single genes, but are also driven by complex DNA rearrangements (deletions, duplications, inversions, insertions, viral integrations, etc.) that are extremely difficult to experimentally trace [35]. However, mostly due to technological constraints, our capacity to precisely characterize the structural nature and mechanisms of numerous putative cancer-causing events such as short-lived complex genomic rearrangements as well as elucidating allele-resolution mutational status has been rather limited. Another significant constraint, which slows down our understanding of tumour evolution, is the lack of suitable experimental in vitro models that could reproduce the ITH of human tumours, whilst mimicking evolutionary forces acting in vivo. For instance, studying evolutionary processes in vitro would require models that would recapitulate the effects of the complex tumour microenvironment, the presence or absence of resources and mimic the space constraints that tumour cells experience whilst developing. On the other side of the spectrum, the task of studying tumour evolution from human patient samples is extremely complex as two major problems can be readily identified: (i) for many cancer types, due to ethical reasons, the biopsies cannot be taken multiple times from the same patient to study the population dynamics of a tumour, and (ii) given that the biopsy is a small fraction of the tumour, it may not necessarily represent the entire heterogeneity of the population. Therefore, most of the current studies trying to understand tumour evolution infer the evolutionary tree using genome profiles that are nothing but a one-off snapshot of a single tumour sample from a single location at a particular point in time, thus limiting our understanding of the population dynamics, complex clonal architecture and the underlying mechanisms of natural selection that indeed shape evolutionary trajectory.

Of course, not everything is bad news. The recent development of liquid biopsies, including circulating tumour cells (CTCs) and circulating tumour nucleic acids (ctDNA) [36,37,38], may help to alleviate, and maybe fully overcome, the above mentioned constraints. Given that these assays require the analysis of blood or other body fluids, they are minimally invasive, thus offering an opportunity to monitor patients in real time on a regular basis, which provides crucial information on tumour development and evolution. Notably, ctDNA extracted from cerebrospinal fluid biopsies have been shown to closely recapitulate the genotypes of tumour biopsy obtained from glioma patients [39]. Similarly, mutations detected when analysing CTCs were consistent with the genetic landscape of bone marrow tumour cells in multiple myeloma patients [40], suggesting that this approach may indeed be a suitable strategy to unravel the extent of tumour heterogeneity. Furthermore, performing comparative genomic studies of CTCs, primary tumours and metastasis in colorectal and metastatic prostate cancer patients has shown that most of the mutations detected in CTCs were also present at sub-clonal level in the primary tumour and metastasis of the same patient [36,41]. Although the mechanisms of CTCs and ctDNA shedding into the bodily fluids is not yet well understood, these data strengthen the possibility that profiling CTCs and ctDNA may allow the efficient identification of the complexity of mutational burden present within primary tumours as well as metastatic lesions. Thus, though technical limitations mainly related to the efficient enrichment in CTCs from liquid biopsy samples still exist, it is reasonable to suggest that applying single cell DNA sequencing approaches to the analysis of CTCs may emerge as a powerful tool to shed light on the dynamics of clonal architecture and the evolutionary processes taking place during tumour progression. Altogether, understanding the mechanisms of tumour development and progression from an evolutionary perspective may have a significant impact on the way anti-cancer therapies are designed and their efficacy further evaluated in clinical settings [42,43,44].

## 3. Models of Tumour Evolution

### 3.1. Origins of Cancer—A Founder Cell

Cancer originates within a tissue ecosystem—a habitat that has evolved over millions of years to enable an overall proper functioning of a multicellular organism, whilst limiting clonal expansion and somatic evolution [45]. This restriction of clonal expansion is achieved through multiple mechanisms including senescence and cell differentiation that limit the number of descendants generated from any given cell. Those mechanisms reduce the number of generations per lineage and consequently reduce the chance of somatic mutations to occur and propagate through clonal expansion.

Therefore, it is thought that under normal conditions, every mechanism that restricts proliferation, neo-angiogenesis, migration/invasion and self-renewal is under tight control. In that regard, the observation of complex and heterogeneous mutational signatures in cancer cells has been suggested to reflect the purposeless evolutionary process that may generate functional solutions (newly arising phenotypes) to overcome the above mentioned constraints [45]. Alterations that generate a phenotype with a selective growth advantage (increased proliferation rate, decreased cell death rate, etc.) are known as “driver” mutations. Driver alterations are defined as genetic alterations that (i) are found in different types of tumours more frequently than it can be expected from a random mutational process, (ii) lead to clonal expansions, (iii) are associated with the molecular processes relevant to oncogenesis and (iv) have been confirmed to cause tumours in functional tests and animal models [35,46,47,48,49,50]. Interestingly, depending on cancer type, most solid tumours require changes in 1 to 10 driver genes to transform a normal cell into a cell with malignant properties (cancer cell) [51,52]. Even though only a small handful of mutations may be sufficient to promote cancer onset, genomics data clearly demonstrate that many more genes, including multiple driver genes, can be mutated in cancer cell populations. These mutations can be clonal or sub-clonal in relation to the alterations that initiate the disease, and reflect evolutionary processes taking place within a tumour. However, not all mutations encountered in cancer cells are linked to “driver” genes. Indeed, hundreds of mutations are selectively neutral and are often propagated alongside driver mutations. Importantly, this particular kind of mutation (known as “passenger” or “hitchhiker” mutations) may not necessarily be neutral throughout the entire progression of the tumour, but may change its impact on tumour evolution in a context dependent manner. For instance, an aberration beneficial for angiogenesis may not be selected at the earliest stages of tumour development, but may be subject to natural selection later on as the tumour microenvironment is depleted from basic resources. Interestingly, by applying an integrative-omics approach to the analysis of gene expression, copy-number alteration and mutation data for in-situ ductal carcinoma, invasive ductal carcinoma and metastatic site samples, it has been suggested that the malignant traits are mostly acquired during early stages of breast cancer and remain constant over its development into the invasive and metastatic disease [53]. Furthermore, it has been proposed that subsequent genetic alterations play a role as “fine-tuning effectors” of the malignant phenotypes established at the early stages of cancer onset.

Interestingly, a long-standing debate in the field has been whether a tumour originates from a single mutated normal cell—a founder cell—or from multiple cells bearing enhanced tumourigenic potential [54,55]. The early stages of cancer development in the context of human tumours cannot be directly observed and therefore the earliest events of cancer onset remain largely unknown. However, the ancestral history of genomic alterations is reflected within cancer cell genomes and thus the information regarding the tumour initiating events may be reconstructed from the patterns of intra-tumour heterogeneity detected in late-stage tumours. Thus, questions regarding the uni- or multicellular nature of cancer origins have been approached by applying a population genetics concept of a “most-recent common ancestor” to the analysis of the multi-regional and single cell whole genome sequencing data. A full set of somatic alterations found in each cell of a cancer cell population represents the most-recent common ancestor or, in other words, the most-recent common genotype. In the phylogenetic tree, this common ancestor represents the shared trunk, while the branches depict the divergent sub-clones. Interestingly, most of the data obtained up to now for different types of cancer generated using multiregional, whole genome, deep sequencing and single cell sequencing suggest the existence of a single “most-recent common ancestor”, arguing that cancer cell populations originate from a single normal cell [3,4,11,16,56,57].

The hypothesis regarding the multi cellular origin of cancer is prevalent for cancers that are caused by exposure to exogenous mutagens (cigarette smoke, ultra violet irradiation) or germ line mutations as well as for multifocal cancers (liver, prostate cancer), suggesting that the so called “field effect” influencing multiple cells simultaneously may take place, leading to more than one cancer initiating cell [58]. Surprisingly, by analysing large cohorts of lung cancer and melanoma samples in which case cigarette smoke and UV can potentially cause the “field effect” due to the widespread nature of tissue exposure, multifocal sequencing revealed that different tumour regions share a common ancestor [4,16,57], suggesting a single-cell origin. Similarly, by sequencing samples obtained from several foci of a multi focal breast cancer, it has been revealed that individual foci were clonally related, thus reinforcing the idea of a single-cell cancer origin [11]. However, in a single case study where single-cell DNA sequencing was used to establish genetic divergence, the authors have proposed that, in that particular case, colon cancer may have a bi-clonal origin [59], offering the possibility that although most of the studies show evidence of a single-cell tumour origin, some cancers may indeed in rare occasions be initiated by more than one mutated cell. Notably, it is important to highlight that the most recent common ancestor may represent a genotype that could have been selected as a result of the last complete selective sweep, leading to the expansion of the most fit sub-clone, and thus might not necessarily point to a cancer inducing genotype/s.

### 3.2. Linear and Neutral Evolution in the Context of Cancer

**Figure 1:**
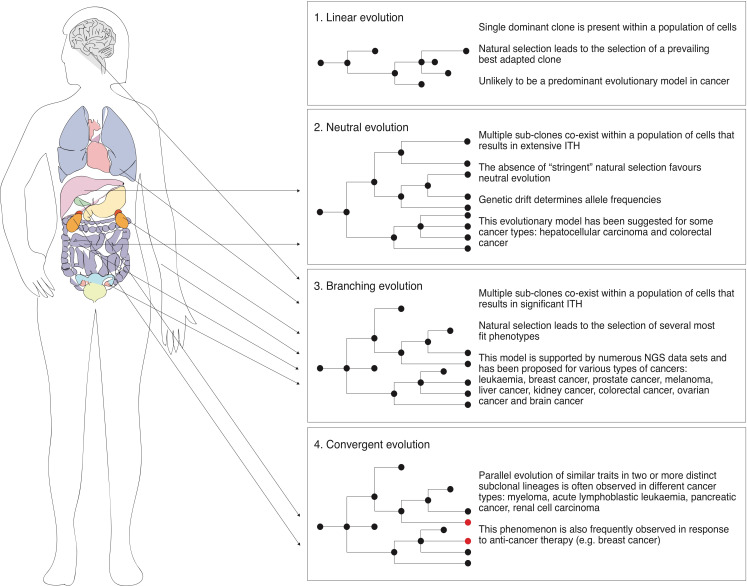
Models of tumour evolution. Scheme summarizing our current knowledge on evolutionary processes taking place in different types of human cancers. Phylogenetic trees depict the relationship among sub-clonal populations over time. Black circles represent nodes of diversification. Red circles depict functional/phenotypic convergence in two genetically distinct sub-clones.

Although the linear tumour evolution model is one of the most accepted in the field, limited convincing experimental data exist to support that this sole model operates during tumour evolution [5]. Strictly following this model, mutations are accumulated in a step-wise manner followed by selective sweeps resulting in a single dominant clone (Figure 1). Consequently, following the basic concepts of this model, selective pressure must occur immediately after a new round of mutations takes place, leading to the selection of a prevailing clone. However, genomic data gathered over the last decade clearly demonstrate extensive ITH in most of the human tumours analysed, thus suggesting the possible coexistence of multiple sub-clones within cancer cell populations [3,4,5,6,7,8,9,10,11,15,16]. Therefore, tumour development is unlikely to be explained exclusively in terms of linear evolution.

Indeed, since significant ITH has been found in tumours of diverse origins, it has been proposed that tumours may follow neutral evolution—a model that was originally proposed in species evolution challenging the Darwinian idea of natural selection. According to this model, there is no selection acting on the tumour along its development, instead, mutations accumulate over time followed by genetic drift—random changes in allele frequencies over generations, that in turn generates extensive ITH. Several pieces of experimental evidence have been proposed in recent years to support non-Darwinian neutral evolution in tumours. In that regard, though being just a case study, it has been shown that the sequencing of 309 spatially distinct regions of a hepatocellular carcinoma from a single patient revealed the coexistence of 20 unique sub-clones [60]. The authors concluded that the clonal diversity of the multiregional sampling showed no evidence of positive Darwinian selection and was consistent with the model of neutral evolution in an expanding population [60]. Furthermore, by analysing the linear relationship between the number of mutations and inverse mutant allele frequency in a large cohort of tumours (904) of different types, Williams et al. suggested that more than 1/3 of these tumours are neutrally evolving [61]. However, the latter study met criticism mainly related to the method the authors developed to draw their conclusions, causing a debate on whether the described patterns of ITH can be explained by neutral evolution [62,63,64,65]. Interestingly, a “Big Bang” model has been proposed for colorectal cancer evolution, whereby extensive ITH arises at the early stages of tumour development, giving rise to multiple sub-clones that are not subject to stringent selection and therefore grow as a single expansion without displaying any individual sub-clone prevalence [66]. In contrast, the model of colorectal cancer evolution proposed by Uchi et al. suggests that, initially, several alterations are accumulated in a stepwise manner, followed by the generation of extensive ITH in the late stages of tumour development tailed by sub-clonal neutral expansion [67]. Overall, these data suggest that in some types of cancer and/or at some stages of cancer progression, natural selection may be rather weak, thus favouring neutral evolution.

### 3.3. Branching Evolution, Parallel Evolution and Convergence

Although some types of cancers may indeed undergo neutral evolution, most of the current NGS data suggest that although the majority of tumours are characterized by significant ITH, selective pressure must be shaping its evolutionary trajectory [3,4,5,6,7,8,9,10,11,15,16,25]. Importantly, natural selection driven by the tumour microenvironment, immune system and competition for space and resources by no means contradicts the coexistence of multiple sub-clones within the cancer cell population. In that regard, a model of branching evolution has been proposed, which suggests that tumour evolution involves the clonal expansion from a founder cell, followed by genetic diversification resulting in multiple clonal lineages evolving in parallel and the eventual clonal selection due to the space/resources competition, various microenvironmental perturbations or exposure to chemotherapeutic agents (Figure 1). Supporting this model, numerous NGS studies, including whole exome, whole genome, multi-regional as well as single-cell DNA sequencing, have reported that branching evolution is most likely to take place in many types of human cancer, including leukaemia, breast cancer, prostate cancer, melanoma, liver cancer, kidney cancer, colorectal cancer, ovarian cancer and brain cancer [4,5,7,8,11,12,17,20,23,57,68,69]. Notably, using single-cell DNA sequencing, some reports suggest that during the branching evolution of tumours, mutations accumulate gradually over time, suggesting multiple rounds of diversification, expansion and selection of the fittest clones [23,70]. In contrast, some studies challenge the paradigm of gradual evolution and suggest that the majority of aberrations occur in punctuated bursts at early stages of tumour onset, after which several dominant clones expand to form a tumour mass [20,71]. Interestingly, Wang et al. reported that aneuploid rearrangements take place at the early stages of tumour evolution and remain stable along the clonal expansion of a tumour, whereas point mutations occur regularly and gradually followed by clonal expansion, leading to extensive sub-clonal diversity [23].

Interestingly, though tumours can originate from different tissues and cell types, a striking consistency can be observed among different types of cancer at the level of essential “Hallmark” traits (unlimited replication, genomic instability, invasion of new niches, etc.), suggesting that some level of “molecular” convergence might operate throughout tumour development [72,73]. In this light, in the classic view of the biological evolution of a species, it is well documented that if two genetically different populations (different species) that share the same ecological niche are subjected to the same selective pressure, the evolutionary processes will be driven towards the same traits that are optimal to survive in this environment, forcing both populations to converge into a certain phenotype, despite harbouring distinct genetic backgrounds [72,74,75]. Therefore, a direct parallel can be drawn between the evolution of the species and cancer biology. Taking this into account, it has been hypothesised that convergent evolution may play a crucial role in cancer development and progression as without convergent evolution there should be no similarities between tumours from different tissues and we would not categorize them into a disease we call “cancer” [72]. Notably, convergence in the context of cancer, which refers to the parallel evolution of similar traits originating from a single clone, is indeed frequently seen within individual tumours (Figure 1) [14,76]. For instance, it has been reported that independent RAS mutations leading to RAS/MAPK pathway activation are present in two distinct-sub-clonal-lineages of myeloma [77]. In another study, independent deletions in PAX5, ETV6 and CDKN2A have been found in distinct sub-clones of acute lymphoblastic leukaemia [78]. Similarly, two overlapping out of frame deletions in exon 6 of PARK2 have been reported to occur in distinct pancreatic cancer metastatic sites [79]. Furthermore, in another example, the analysis of 14 specimens of renal cell carcinoma from 5 patients has revealed that 13 of 14 specimens showed functionally convergent alterations with an activating effect on mTOR signalling [80].

Importantly, the parallel evolution of similar traits has also been frequently observed following anti-cancer therapy [42,81,82]. For instance, it has been reported that the analysis of samples obtained from 14 metastatic sites of a breast cancer patient who previously received and developed resistance to PI(3)Ka inhibitor (BYL719) displayed single copy loss of PTEN in all metastatic lesions when compared to the primary pre-treatment tumour and peri-aortic lesions [83]. Interestingly, 10 out of 14 metastatic lesions analysed harboured additional genomic alterations in PTEN. Similar results have been obtained from an additional cohort of breast cancer patients that developed resistance to BYL719. Overall, these data suggest that PTEN underwent a parallel loss in distinct metastatic sites upon selective therapeutic pressure [83]. Similarly, multiple de novo KRAS mutations were detected in circulating tumour DNA obtained from patients that developed resistance to the EGFR monoclonal antibody therapy [82], suggesting the independent evolution of functionally similar traits, in this case resistance to a particular therapeutic approach.

Importantly, although most of the studies reported to date seem to focus on a single model of evolution, accumulating evidence suggests that these models are not mutually exclusive and may be operating at different stages of tumour initiation and progression. For instance, whilst linear evolution has not been experimentally supported as a model of cancer evolution, it has been suggested that it may take place at the earliest stages of cancer initiation followed by branching evolution as the population expands [4,5,7,8,11,12,17,20,23,57,68,69]. Moreover, it is possible to hypothesise that neutral evolution and branching evolution that involves natural selection may take place within the same tumour, but at different stages of its development. In this light, the co-existence of multiple sub-clones within a single tumour could be explained by the accumulation of mutations within different sub-clones that are indeed evolutionarily neutral and therefore not selected in the first instance. However, those sub-clones may eventually be subjected to natural selection, thus resulting in pervasive changes in sub-clonal architecture. Along those lines, at the earliest stages of tumour development the competition for space and resources may be minimal, thus favouring neutral evolution, whereas at later stages of tumour development—as the population size increases—branching evolution involving natural selection may take over and become the driving evolutionary mechanism.

## 4. Non-Genetic Mechanisms in Cancer Progression and Adaptation

Although most studies address cancer evolution from a genetic perspective, it is becoming increasingly evident that cancer initiation, progression and adaptation may be fuelled by non-genetic mechanisms, thus suggesting that cancer may also evolve in a mutation-independent manner [32,84]. Non-genetic mechanisms (histones and DNA modifications, imprinting, paramutation, posttranslational gene silencing, etc.) can lead to the establishment of metastable and stable phenotypic states that can be inherited upon cell division and propagated through many cell generations. Perhaps the best example of this phenomenon is the metazoan body, in which a single genome generates a vast array of stable functionally distinct cellular phenotypes. This should prompt us to think that the establishment of “cancer” phenotypes does not necessarily have to be driven by a genetic component and that non-genetic mechanisms can indeed be a major contributor to the development of malignant traits resulting in cancer initiation, progression and dissemination. Indeed, multiple studies have shown that tumour initiation and dissemination can be driven by non-genetic programs [85,86,87,88]. These examples suggest that the genetic component may not be absolutely necessary for cancer development and progression and that non-genetic mechanisms might play a crucial role in cancer origin. Furthermore, it has long been observed and is now being solidified with the advent of single-cell RNA sequencing (scRNA-seq) that independently of genetic heterogeneity, cancer cell populations display significant non-genetic variability [30,31,89,90,91,92,93], indicating that cancer cells bear the capacity to generate distinct states leading to a coexisting spectrum of metastable states upon which Darwinian as well as Lamarckian evolution may act.

### 4.1. Molecular Basis of Non-Genetic Heterogeneity

**Figure 2:**
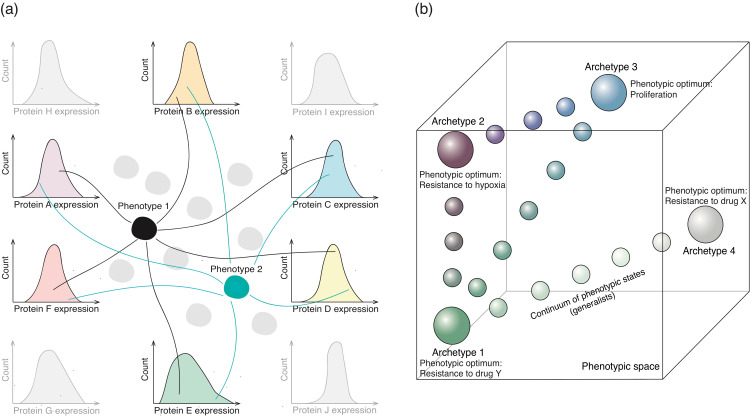
Non-genetic heterogeneity within clonal populations of cells. (**a**) Flow cytometry analysis reveals that cells within a clonal population display divergence in protein expression levels (normal distribution), which results in the establishment of distinct phenotypes. (**b**) Scheme representing a model [96] that suggests that cells within a population may occupy distinct positions in a “gene expression space”. Depending on the position within the “gene expression space” and the characteristics of the microenvironment, some of those cells will represent specialized phenotypes—archetypes (in the figure: Archetype 1, Archetype 2, Archetype 3, Archetype 4) that display a “functional optimum” at performing defined tasks (proliferation, drug resistance, hypoxia resistance, etc.) in the present environment. The model also proposes that a number of intermediate phenotypes may exist and reside in the phenotypic space between specialized archetypes. Those phenotypes are called generalists. It is suggested that generalists are less efficient at performing specialized tasks; however, they may have a survival advantage in rapidly changing environments.

Non-genetic heterogeneity can be readily observed in clonal populations of cells. In fact, flow cytometry analysis of the abundance of perhaps any protein results in a bell-shaped histogram, evidencing that protein expression levels within a population of genetically identical cells largely vary in a cell-to-cell manner (Figure 2a). It has been suggested that such variations in expression levels can be either accounted for fast short-lasting fluctuations, or be a result of relatively stable variability caused by slow fluctuations in gene expression patterns [32]. Indeed, it has been demonstrated that the expression levels of different genes exhibit slow fluctuations in mammalian cells [94]. For example, when multipotent blood progenitor cells are sorted according to the highest and lowest expression levels of SCA1 (stem cell antigen 1), it is only after 10 days that either of these populations regenerate the previously observed bell-shaped distribution of SCA1 expression [95]. These data argue against fast fluctuations in gene expression patterns and suggest that some sort of molecular memory that maintains a rather defined expression level for extended periods of time exists. Notably, cells displaying strikingly different levels of SCA1 expression were also characterized by genome-wide differences in gene expression profiles, thus suggesting the coexistence of distinct phenotypic states (Figure 2a) [95].

The establishment of distinct phenotypic states can be mediated by multiple stochastic events that are driven by cell extrinsic, cell intrinsic and allele-specific variations [97,98] (Appendix A.1). These variations may arise as a result of differential histone and DNA modifications [99,100,101,102], variable 3D genome architecture [97,98], the dynamic binding of the transcriptional machinery [103], dynamic interactions among regulatory chromatin regions (e.g., olfactory receptors) [104,105,106], as well as the asymmetric redistribution of crucial cellular components upon cell division, such as coding and non-coding RNAs as well as proteins involved in the regulation of gene expression and in various signalling pathways [107]. Although the mechanisms that enable the stabilization and maintenance of phenotypic states are poorly understood, it has been suggested that a stable state may represent a so called “attractor state”—a minimum energy equilibrium state [108]. However, multiple recent experimental studies demonstrate that clonal populations of somatic cells do not represent a single phenotypic state but instead exhibit significant non-genetic heterogeneity, suggesting that there may be multiple states coexisting within an isogenic population of cells [33,89,91,109]. Notably, experimental data show that once a certain phenotype is separated from a phenotypically heterogeneous population (for example, outliers of SCA1 expression [95]), it will eventually reconstitute the pre-existing heterogeneity of the population, suggesting that phenotypic states observed within clonal populations of somatic cells are characterized by a plastic capacity to transition from one state to another [90,95]. Altogether, it is possible to hypothesise that isogenic populations of somatic cells consist of several metastable “sub-attractor states” that are capable of undergoing phenotypic switching. Importantly, phenotypic states within clonal populations of cells may play a crucial role in population behaviour, survival and dynamics, as they display a wide range of differential responses to environmental cues and can undergo different cell fates. For instance, it has been reported that SCA1 outliers commit to different lineages when undergoing differentiation [95].

Taking this into account, and given that phenotypic states can be inherited over many cell generations and therefore be sustained for prolonged periods of time, the idea that non-genetic heterogeneity constitutes a substrate for natural selection suggests that it can indeed impact the evolutionary course of a tumour.

### 4.2. Drug-Tolerant Phenotypic States

**Figure 3:**
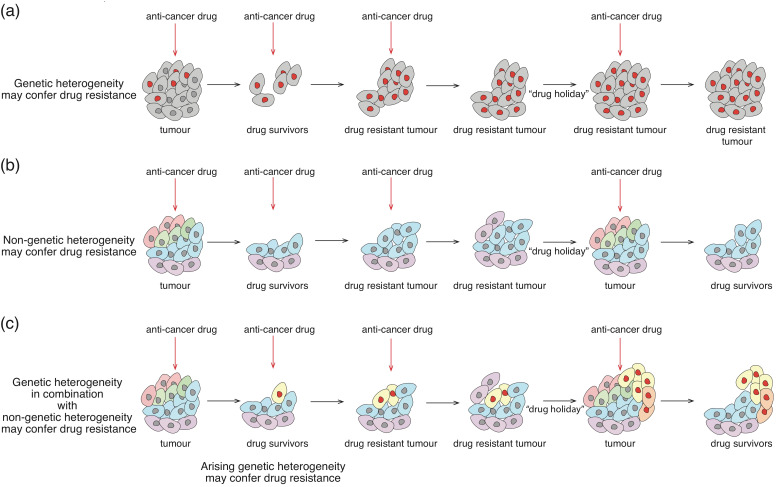
Models of cancer progression upon drug treatment. (**a**) A population of cancer cells may contain a pre-existing genetic mutation that confers resistance to an anti-cancer drug. This genetically encoded phenotype is further selected upon drug treatment, leading to outgrowth of a drug-resistant tumour. (**b**) A population of cancer cells may display extensive non-genetic heterogeneity resulting in multiple phenotypic (epi-)states co-existing within a population. Some of those (epi-)states may be resistant to drug treatment. Non-genetically encoded phenotypes can be further selected, leading to an outgrowth of a drug-resistant tumour. However, contrary to genetically encoded resistance, when drug treatment is discontinued, the population of cancer cells may re-establish its initial heterogeneity that displays drug sensitive phenotypes. (**c**) A population of cancer cells may contain drug resistant phenotypes driven by both genetic and non-genetic mechanisms. Such resistant phenotypes are selected upon drug treatment, leading to the generation of drug-resistant tumours. Each genotype may produce a variety of phenotypic (epi-)states. The colour of the nuclei depicts a defined genotype; the colour of the cytoplasm shows distinct phenotypes that may result from genetic and non-genetic mechanisms.

Indeed, it has been largely accepted that genetically distinct sub-clones within a cancer cell population display a differential response to anti-tumour drugs and are believed to be the main source of drug-resistance. However, the phenomenon of reversible resistance, often observed in clinical settings [110], cannot be explained by genetic variations and suggests that drug resistance goes beyond genetic heterogeneity (Figure 3a–c). Along those lines, it has been shown that non-small cell lung carcinoma patients display an initial efficient response to an EGFR tyrosine kinase inhibitor; however, upon continuous administration, patients rapidly develop resistance to this drug. Surprisingly, if the drug treatment is discontinued and the patients that developed resistance to this therapeutic paradigm are subjected to a “drug holiday” (drug removal from treatment), the sensitivity to the treatment can be regained [111,112]. This observation cannot be explained by a drug-mediated selection of genetic clones that are resistant to the EGFR tyrosine kinase inhibitors (gefitinib and erlotinib) [33], and argues in favour of the acquisition of a non-genetically driven reversible drug tolerant state. Interestingly, a comparative analysis of drug sensitive versus drug tolerant cells revealed drastic differences in the overall gene expression patterns, thus suggesting major chromatin re-arrangements. Indeed, it has been found that the histone demethylase KDM5A, known to exhibit histone H3K4 demethylating activity, is essential for the establishment of a metastable state that harbours drug-tolerant phenotypes [33]. Furthermore, it has been reported that reversible drug tolerant states identified in different cancer cell lines of various origin (melanoma, lung, breast and colon cancer) are characterized by consistent genome wide changes in histone modifications (an increase in H3K9me3, and decrease in H3K4me3 and H3K27me3) that were followed by altered gene expression patterns [113]. Strikingly, the observed epigenome alterations/changes were reversible upon a period of drug holiday. Notably, a crucial role of chromatin regulators in driving reversible drug resistant phenotypes has also been reported for T-cell acute lymphoblastic leukaemia [114]. In this study, BRD4—a member of the BET family of bromodomains that bind acetylated histones—has been found to play an essential role in the maintenance of a drug “persistor” state, presumably through targeting genes required for cell proliferation. Overall, these examples suggest that changes in chromatin architecture may represent a key adaptive mechanism that drives the establishment and maintenance of metastable drug resistant states, enabling dynamic cancer cell behaviour in response to harsh environmental conditions.

Importantly, the phenomenon of reversible resistance has been observed in multiple studies using clonal populations of cancer cells in cell culture settings, in which genetic heterogeneity can be easily excluded [33,34,89,109,115,116,117]. Interestingly, it has been demonstrated that when clonal populations of cancer cells are exposed to the cytotoxic cytokine TRAIL (Tumour Necrosis Factor Related Apoptosis Inducing Ligand), only a certain fraction of the population undergoes cell death, whilst the rest of the population survives, continues proliferating and acquires resistance upon repetitive exposure to the ligand. Interestingly, when TRAIL treatment is discontinued, cells maintain a drug-tolerant state for a brief period of time but eventually revert from their resistant phenotype and regain the initial sensitivity to TRAIL-induced apoptosis within several cell divisions [34,109]. These results support the notion that isogenic populations of cells exhibit extensive non-genetic heterogeneity capable of supporting striking divergence in cell fate in response to environmental cues. Furthermore, it has been reported that among the cells that die, the time between TRAIL exposure and caspase activation significantly varies from one cell to another [118], highlighting the complexity of non-genetic mechanisms of variations that may occur at multiple levels of the cellular response to a given cue.

Importantly, it has been recently demonstrated that breast cancer cells exhibit extensive non-genetically driven metabolic heterogeneity [119]. Similarly to the aforementioned example, identified metabolic states are heritable upon mitosis and can be propagated for multiple generations. Furthermore, it was shown that cells with high and low intracellular glucose levels (high-glucose and low-glucose, respectively) could switch their metabolic state. However, it appears that high-glucose cells may reconstitute the initial metabolic heterogeneity more rapidly as compared to low-glucose cells, suggesting that different states may bear variable plastic potential. Moreover, high-glucose cells are highly dependent on extra-cellular pyruvate as compared to low-glucose cells, evidencing that distinct metabolic states may undergo differential responses to various environmental cues. This variability may be beneficial in a rapidly changing tumour microenvironment, including proliferation advantage and resistance to anti-cancer drugs. Indeed, increased levels of pyruvate in some of the spatially separated histological sections obtained from primary human kidney tumours support the growth of primary kidney cancer cells, suggesting an important role of the metabolic component in population dynamics [120]. Interestingly, it has also been shown that glutamine deficiency observed in core regions of melanoma xenografts drives H3K27 methylation, which results in cancer cell resistance to BRAF inhibitors [121]. These data reinforce the notion that a highly variable metabolic environment may promote intra-tumour heterogeneity, which in turn may play a crucial role in the way a tumour responds as a whole to anti-cancer therapies.

Overall, the above observations suggest that cancer cell survival in hostile environments (such as exposure to drugs) and their adaptation to a challenging environment do not necessarily require a genetic component, but may be governed by non-genetic mechanisms. In this context, cellular persistence or transient resistance may either arise due to the selection of a pre-existing refractory phenotype or due to the drug-mediated induction of a resistant phenotype. Importantly, both scenarios are not mutually exclusive and might operate concomitantly. Interestingly, it has been proposed that in this context, Darwinian and Lamarckian evolution may come into play together as it is hypothesised that non-genetic heterogeneity may provide cancer cell populations with the phenotypic state/s capable of withstanding an initial exposure to the drug (Darwinian natural selection) and may further prime drug-induced complex adaptive mechanisms upon continued exposure to the therapeutic agent (Lamarckian induction-mediated adaptation; [84]).

### 4.3. EMT at the Basis of Tumour-Initiating Phenotype and Tumour Dissemination

The critical role of transient metastable states in cancer evolution is not limited to drug resistant phenotypes. Indeed, numerous pieces of evidence suggest that the ability of cancer cells to disseminate and populate new niches is driven by a transient non-genetic program known as the epithelial-to-mesenchymal transition (EMT). EMT is a complex-highly dynamic-reversible multi step process (Appendix A.2) that involves gradual changes in a number of cell properties (morphology, cell polarization, motility, invasive potential, stem-like properties, etc.), resulting in a transition from an epithelial to a mesenchymal phenotype. This leads to the remodelling of cell-to-cell and cell-to-extracellular matrix interactions, which in turn results in cells’ detachment from each other and the underlying basement membrane. Notably, cells rarely reach “full transition” and, therefore, EMT should not be viewed as a binary phenotypic switch, but rather as a process that generates a spectrum of transient quasi-mesenchymal phenotypic states [122,123,124,125,126]. Cells residing in quasi-mesenchymal states can undergo the reverse process known as the mesenchymal-to-epithelial transition (MET) (Appendix A.2), generating cells exerting an epithelial phenotype, thus highlighting the plastic nature of these transitions [127].

Notably, the EMT transition has been proposed to be a major event that initiates the invasion and dissemination cascade in primary carcinomas, including those arising in the breast, prostate, colon, head and neck, ovary and lung [128], thus suggesting that at least the initial stages of metastatic spread may be induced in a mutation-independent manner [122], highlighting a crucial role for non-genetic programs in cancer progression [129]. Supporting the role of non-genetic programs at the origin of metastatic phenotypes, a wealth of evidence suggests that invasive tumours express increased levels of EMT related transcription factors (EMT-TFs). For instance, it has been demonstrated that ZEB1 and SNAIL expression, transcription factors activated during the EMT transition, are essential for invasion and metastasis in mouse carcinoma models [130,131]. Moreover, it has been shown that the increased expression of SLUG (an EMT-TF) results in a metastatic phenotype in otherwise non-metastatic cells, suggesting a pivotal role for EMT in cancer dissemination [132]. Interestingly, several observations indicate that disseminated cells displaying “more” mesenchymal phenotype switch back to a “more” epithelial phenotype upon residing in a new tissue. Thus, the existing model of MET suggests that cells undergo a reverse transition as a direct consequence of the absence of extracellular EMT-promoting signals encountered mainly within its niche of origin, therefore leading to changes in epigenetic mechanisms that control the repression of epithelial traits, which in turn leads to the re-activation of epithelial markers associated with rapid proliferative capabilities [133,134,135,136]. Therefore, the dynamic and reversible nature of EMT may facilitate multiple crucial events along the progression of cancer.

Interestingly, it has been suggested that EMT generates cells that exhibit stem-like properties, often referred to as cancer stem cells (CSCs)—tumour-initiating cells that upon rounds of symmetric and asymmetric cell division can reproduce themselves (self-renewal) and give rise to a bulk of cells that do not exert tumourigenic potential [86,132,137,138,139]. Along those lines, it has been shown that populations of cells that do not exhibit stem-like properties undergo EMT under certain conditions, which is associated with the acquisition of CSC markers and increased capacity to generate tumours in mice [130,139,140]. These cells have been shown to display an intermediate phenotype that lies at a “sweet spot” along the epithelial-to-mesenchymal transition spectrum; however, the mechanisms that stabilize the cells in that quasi-mesenchymal state remain largely elusive [123,141].

As the above-mentioned data clearly show that cancer cells display extensive phenotypic plasticity, it is possible to hypothesise that EMT/MET-like processes (i) may allow the establishment of metastable states and further facilitate the survival and fast adaptation of cancer cell populations in response to changing environments, and (ii) could play a crucial role in cancer progression by promoting the emergence of tumour-initiating phenotypes. Taken together, the observations listed above reinforce the idea that evolutionary processes such as natural selection and adaptation in the context of cancer might act not only upon stable heritable genetic alterations, but may also operate through non-genetically encoded phenotypic states.

### 4.4. Phenotypic Plasticity

Phenotypic plasticity in response to environmental cues is a phenomenon well documented for a large variety of organisms. A spectrum of phenotypic variations that can be produced by a single genotype exposed to different environments is known as a “reaction norm”. Reaction norms allow organisms to quickly adapt to the changes in the environment and efficiently maximize their fitness under the given circumstances without changes in the genome. A well-known example of such phenotypic plasticity is described in the protist *Tetrahymena vorax*, which is able to switch between two distinct morphotypes—macrostome and microstome—in response to environmental changes, such as the relative abundance of bacteria versus other protists [142,143]. Interestingly, when a macrostome or microstome undergoes cell division it can either maintain its corresponding morphology or change to a distinct morphotype depending on resource abundance. Therefore, phenotypic plasticity may create a wide range of environmentally contingent traits that grant flexible behaviour in response to frequently changing conditions. Considering the highly variable nature of the tumour microenvironment, it is not surprising that tumours also display flexible behaviour. For instance, it has been shown that oxygenation within the tumour mass is constantly changing [144], suggesting extensive spatial and temporal variation of pO2 (partial pressure of oxygen) and nutrient delivery to the same tumour region [145]. These observations suggest that cancer cells may be exposed to frequently and rapidly changing environments, which makes it reasonable to hypothesise that non-genetic mechanisms of adaptation must exist to support cancer cell survival in rapidly fluctuating conditions. Along those lines, several studies have demonstrated that cancer cells can be driven towards more or less aggressive phenotypes depending on the complexity of their microenvironment. In that regard, it was found that inflammation facilitates tumour progression and leads to decreased survival time in mice models, while reduced inflammation has the opposite effect [146,147], thus suggesting that changes in the microenvironment composition may promote cancer cell phenotypic diversification, which in turn shifts the evolutionary trajectory of a tumour.

Given the complex ecological niche and its fast-paced environment, cancer cell populations must be able to multi-task in order to sustain their survival. However, not a single biological system can excel at multiple tasks at the same time. Therefore, molecular mechanisms that would allow the establishment of balance in order to thrive must exist. In that regard, it has been suggested that cancer cell populations may face a trade-off between different tasks [96,148,149,150]. Interestingly, according to this model, a cell that is “best” at a certain task, for instance proliferation, represents a specialized archetype (Figure 2b) [96]. The archetypes for distinct tasks are different, as they require distinct properties (e.g., gene expression profiles) to be optimal at performing a given function. The theorem also suggests that there are phenotypes that lie between specialized archetypes in a gene expression space, known as “generalists” that are suggested to be less optimal at performing a specialized task, but have better survival chances in an ever-changing environment. The most optimal and beneficial position within that space depends on the most important task at that particular moment; however, the importance of the task may rapidly change, suggesting that a specialized archetype may not be the most beneficial in a fast-paced environment. It follows that phenotypic plasticity might be a necessary feature required to enable the survival of cancer cell populations in complex microenvironments. In that regard, non-genetic heterogeneity observed within isogenic populations of cancer cells prompts us to hypothesise that each genetic sub-clone within a tumour may generate multiple phenotypically distinct sub-populations. This, in turn, may create a continuum of “generalist” states distributed within the gene expression space at different distances from specialized archetypes, which may significantly increase the capacity of a population to efficiently adapt to changing conditions. Given that tumours are generally not spatially homogeneous, a continuum of states might maximize general tumour performance, providing versatile solutions for various possible environmental exposures. Altogether, the aforementioned concepts suggest that non-genetic heterogeneity may serve as a mechanism of mutation-less evolution and support the rapid adaptation of cancer cell populations to changing conditions.

**Figure 4:**
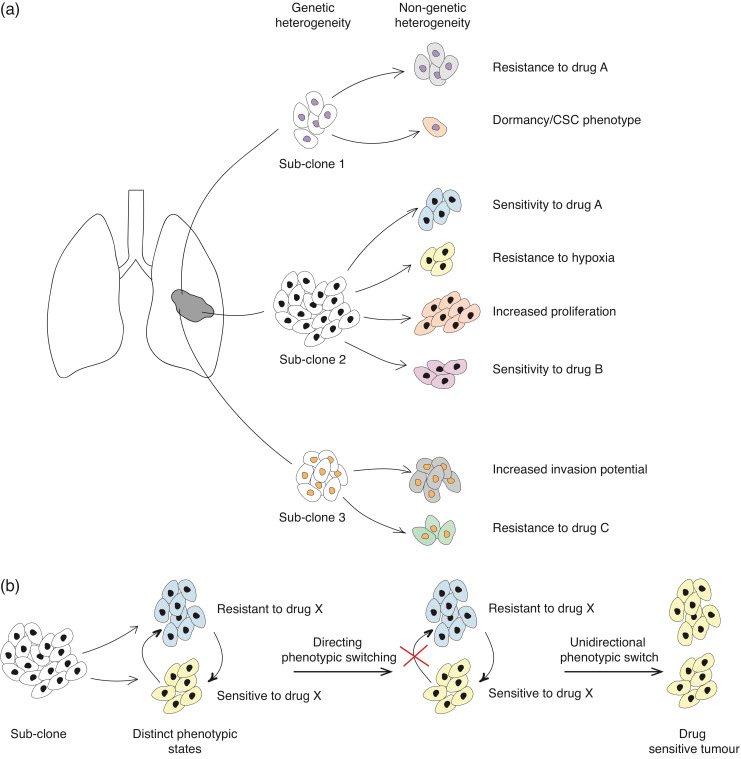
Genetic and non-genetic heterogeneity and their functional relevance in cancer evolution. (**a**) Cancer cell populations may be characterized by extensive genetic and non-genetic heterogeneity, which leads to a variety of phenotypic states with distinct functional characteristics. This may have a crucial impact on cancer progression, growth rates, metastatic potential, resistance to immune surveillance, response to drugs, etc. (**b**) A single genotype may give rise to cells that display functionally distinct phenotypes that will respond differently to various biological cues (for example, anti-cancer drugs). Understanding the molecular basis orchestrating the establishment and maintenance of such phenotypic states may provide a tool to selectively direct the development of cancer cell populations towards less aggressive drug sensitive phenotypes. The colour of the nuclei depicts a defined genotype; the colour of the cytoplasm shows distinct phenotypes that may result from genetic and non-genetic mechanisms.

In light of this, the fact that phenotypic states may change over time and are often reversible in nature may allow cancer cells to withstand harsh conditions and survive selective environments that may also be temporal. Notably, many of the aforementioned examples suggest that phenotypic states established either prior to or upon induction can be maintained if the environment remains stable. This can be potentially achieved by two different mechanisms. In the first one, phenotypic plasticity may reconstruct the response at every cell generation, allowing the population to survive and resulting in a constant frequency of a phenotypic variant as long as the environmental cue persists. However, the phenotype is not intrinsically inherited and is lost shortly after the removal of the stimulus [114,151,152]. In the second scenario, prolonged exposures to a certain condition may lead to a temporal loss of plasticity, generating a rather stable phenotypic variant that may be inherited in multiple generations and maintained in the absence of an environmental cue [89,153,154]. Interestingly, several studies on transgenerational inheritance in multicellular organisms have demonstrated that the number of generations exposed to a stimulus positively correlates with the strength and duration (“memory”) of non-genetic effects [155,156,157], suggesting a gradual stabilization of a phenotype upon which selection can act. Considering both scenarios, it is possible to suggest that from the evolutionary perspective, the acquisition of phenotypic states can be viewed as a gain or loss of function, which in turn enables evolutionary processes such as selection and adaptation to take place. Importantly, non-genetic variants may potentially lead to genetic assimilation—a process by which a phenotype induced in response to an environmental cue becomes genetically encoded and makes the phenotype constitutive. Although a constitutive phenotype may be less costly than a plastic one, it is possible to hypothesise that constitutive phenotypes are beneficial in stable environments, whilst being maladaptive in rapidly changing environments. Though it remains to be demonstrated, we voice the opinion that the acquisition of genetic mutation should not be viewed as a process that will flatten the plastic reaction norm, in contrast, a new genotype may result in a variety of new phenotypic states (Figure 4a). Therefore, phenotypic plasticity that leads to the establishment of non-genetic heterogeneity, and may govern adaptive processes, adds another level of complexity to our understanding of tumour evolution and therefore should not be neglected.

## 5. Cancer Ecosystem

### 5.1. Microenvironment—The Main Source of Selective Pressure

Tumours are not just independent masses of transformed cells; instead, cancer cell populations reside within a very complex multifactorial microenvironment composed of non-cellular (extracellular matrix) and cellular components. Besides being surrounded by the normal cells of the niche of origin, many other types of cells are recruited to the tumour mass, such as T and B lymphocytes, natural killers (NK) and natural killer T cells (NKT cells), tumour-associated macrophages, dendritic cells, myeloid cells, adipocytes, cancer-associated fibroblasts, tumour associated neutrophils, vascular endothelial cells, pericytes and lymphatic endothelial cells. Importantly, cancer must be viewed as an open system, where cancer cells interact with normal ones altering the local environment. These interactions may affect tumour development by establishing tumour-promoting or tumour-suppressing environments often driven by cytokines, chemokines, growth factors, inflammatory and matrix remodelling enzymes. These complex and dynamic interactions between malignant and non-malignant cells, as well as extracellular matrix and mechanical factors, represent the main source of selective pressure and therefore are major drivers of natural selection.

### 5.2. Extracellular Matrix in Tumour Progression

The extracellular matrix (ECM) is mainly composed of collagens, fibronectin, proteoglycans, laminins and elastin and is the non-cellular component of any tissue that provides both physical (structural) and biochemical support to cells. It is largely believed that the ECM is responsible for cell-to-cell communication, cell proliferation, motility and adhesion [158] and plays a fundamental role in organ architecture in metazoans. “Cancer tissue” is not an exception as tumours often display desmoplasia—a fibrotic state that is characterized by the extensive deposition of ECM by cancer cells themselves and to an even greater extent by carcinoma associated fibroblasts that are found in all solid tumours [158]. Notably, the ECM of the tumour microenvironment significantly differs in its physical characteristics when compared to normal tissue. Indeed, the ECM found in tumours is much more abundant, stiffer and denser. These properties as well as the altered biochemical composition of the tumour ECM find a direct correlation with poor clinical prognosis. Hence, it has been suggested that this may be a direct consequence of multiple altered ECM-mediated processes, such as sustaining proliferative signalling, evading growth suppressors, inducing angiogenesis, avoiding immune response, leading to metabolic reprogramming and having negative effects on the efficacy of anti-cancer therapy including radio-, immuno- and chemotherapy [159]. Therefore, cues provided by the ECM influence the behaviour of cancer cell populations and appear to be critical in supporting malignancy (Table 1).

Importantly, the ECM can promote non-genetic heterogeneity within a tumour, which in turn may guide tumour development towards more aggressive phenotypes. In that regard, it has been demonstrated that increased stiffness of the ECM promotes the translocation of EMT-regulating transcription factors into the nucleus and drives EMT in breast cancer and pancreatic ductal adenocarcinoma (PDAC) [160,161]. As discussed in the previous sections of this review, EMT may further orchestrate invasion and metastatic dissemination [140]. Furthermore, ECM remodelling may drive rapid phenotypic changes that confer resistance to anti-cancer therapy. Interestingly, it has been demonstrated that PLX4720—a BRAF inhibitor used to treat BRAF-mutated melanoma cells—leads to the activation of melanoma associated fibroblasts causing ECM remodelling, which triggers increased integrin β1/FAK/Scr signalling in melanoma cells. This is followed by ERK signalling activation that altogether leads to the resistance of melanoma cells to PLX4720, which can be circumvented by the inhibition of BRAF and FAK [162]. Similarly, it has been suggested that BRAF inhibitors induce cancer cell mediated fibroblast differentiation, followed by fibronectin expression leading to AKT/PI3K activation, which abrogates the cytotoxic response to BRAF inhibitors [163]. Thus, the ECM has a profound role in modulating intra-tumour population dynamics by driving phenotypic changes that may promote metastatic disease and facilitate adaptations taking place upon drug treatment, suggesting its crucial role in shaping the evolutionary path of cancer. Further supporting this notion, ECM components may promote genetic instability [186], which might lead to changes in the genetic landscape of cancer cell populations, contributing to genetic heterogeneity.

### 5.3. Immune Cell Component of the Tumour Microenvironment

The cellular components within the tumour microenvironment can modulate an immune response by either favouring or suppressing tumour growth, thus having direct effects on the dynamics of the cancer cell population. The anti-tumour response is mainly mediated by CD8 cytotoxic T lymphocytes (CTL) that recognise the major histocompatibility complex I (MHCI) antigens expressed on the surface of tumour cells and induce cancer cell death. However, cancer cells and the tumour microenvironment can effectively suppress the immune response, making this natural anti-cancer defence mechanism largely ineffective in the majority of cases. Notably, it has been widely accepted that the strategies which cancer cells utilize to evade immune responses can be a result of a positive selection of a sub-population of tumour cells that does not express tumour associated antigens (TAA) recognised by CTL. Moreover, tumour cells may secrete cytokines and chemokines (TGF-β, IL-6, IL-10) that can inhibit immune responses at multiple levels and promote phenotypic changes towards more aggressive phenotypes [122,167,187,188].

Lately, it has become increasingly evident that the inflammatory environment often observed in different types of tumours plays a crucial role in cancer onset and progression through a complex network of interactions between cancer and inflammatory cells (Table 1). In that regard, it has been suggested that in response to hypoxia and necrosis, cancer cells and the tumour microenvironment produce cytokines that attract monocytes and macrophages that induce the expression of a panel of tumour promoting factors [168,169]. Along those lines, it has been shown that MAPK–inhibitors used for the treatment of BRAF-mutant melanomas induce TNF-α production by a macrophage population that triggers the expression of MITF (microphthalmia transcription factor), resulting in the resistance of cancer cells to MAPK inhibitors [170]. Furthermore, besides macrophages, neutrophils can also support tumour progression through several mechanisms, such as the production of tumour-promoting growth factors as well as maintaining inflammation. Overall, the inflammatory environment may promote tumour growth either directly by inducing cancer cell proliferation, or indirectly by down-modulating the immune response, activating tumour-promoting innate immunity signalling, impairing the induction of angiogenesis and removing constrains in tissue remodelling which, in turn, will favour tumour dissemination [167].

Interestingly, it has been recently demonstrated that the immune landscape of the tumour microenvironment in triple negative breast cancer has a profound impact on therapeutic outcome [171]. In that regard, Kim et al. identified three subtypes of triple negative breast cancer in relation to the composition of the immune population: (1) macrophage-enriched subtype (MES) with few neutrophils, (ii) neutrophil-enriched subtype (NES), and (iii) immunological cold subtype with scarce immune infiltrate. Importantly, it was suggested that NES and cold subtypes are resistant to immunotherapy, whereas MES shows a range of sensitive phenotypes. The authors also reported that the same tumour could give rise to different immune subtypes, thus suggesting that distinct subtypes may coexist within the same tumour [171]. Along those lines, by performing multiregional analysis of tumours obtained from 85 patients, it has been recently reported that lung adenocarcinoma and lung squamous cell carcinoma are also characterized by high geospatial immune variability between different regions of the same tumour [189]. Interestingly, genetic divergence in cancer differs according to the immune subtype, with significantly lower genomic distances in immune cold regions as compared to immune hot regions. Furthermore, dominant sub-clones in pairs of immune cold regions were more closely related on the phylogenetic tree, while dominant clones of immune hot regions seem to diversify earlier in the phylogenetic history. Overall, this study suggests that the immune microenvironment may contribute to the micro evolutionary processes taking place within the tumour mass. Notably, patients with a high number of immune cold regions (>1) were at higher risk of relapse when compared to those with a low number of immune cold (≤1) regions, reinforcing the crucial role of the tumour microenvironment in cancer progression [189]. Similarly, by analysing multifocal biopsies from 15 non-small cell lung cancer patients, Jia et al. revealed that intratumour immune spatial heterogeneity was as high as inter-patient variation [190], further highlighting the complexity of individual tumour ecological niches. Altogether, these important findings suggest that intra-tumour heterogeneity goes far beyond the simplified cell-centric view of genetic and non-genetic variability among cancer cells. Indeed, cancer cell populations may reside within remarkably complex and spatially heterogeneous ecological niches, which can drive distinct evolutionary trajectories within individual cancer cell populations contributing to extensive genetic and non-genetic intra-tumour heterogeneity. On the other hand, genetically and non-genetically diverse sub-clones might also modify their local microenvironment [171,191]. Taken together, these reciprocal interactions can have a profound impact on natural selection and adaptive mechanisms along tumour evolution and must be considered when evaluating tumour progression and therapy.

### 5.4. Niche Construction

Notably, interactions among tumour cells and their ecological niche are often nonlinear and bidirectional, which suggests that environmental pressures select cancer cells best adapted to this particular local microenvironment. However, cancer cells also constantly change/adapt their surrounding microenvironment to better suit their needs. In evolutionary biology, this strategy is known as “niche construction” and has been proposed to govern the coevolution of cancer cell populations and their ecological niche [192]. Perhaps the best example of such coevolution is intra-tumour hypoxia, which is a result of limited O2 delivery to intricate, deep-buried tumour cells within the tumour mass [193]. In this context, reduced oxygen concentrations trigger the activation of gene expression programs mediated by hypoxia-inducible transcription factors (HIFs), leading to an increased activation of pathways that facilitate the survival of cells that are better adapted to acidic conditions and inducing invasive tumour growth. Importantly, the accumulation of HIF-1 alpha leads to the expression of VEGF (vascular endothelial growth factor), which promotes vascularization in hypoxic regions [164]. Moreover, HIF-1 expression reduces immune responses by suppressing CD8+ T cell activation. This example clearly demonstrates the interplay between the tumour microenvironment and the evolution of the cancer cell population in response to challenging environmental conditions, and highlights the capacity of tumour cells to modify their niche in a way that favours tumour progression [165]. Notably, it has been reported that the hypoxic response can be induced under normoxic conditions, suggesting that although initially activated in response to limited O2 delivery to tumour cells, the strategy can provide an adaptive advantage even in the absence of the initial trigger, thus highlighting its evolutionary significance [192]. Along those lines, harsh environmental conditions such as hypoxia, acidosis, low-glucose and starvation have all been shown to select cells that exhibit increased rates of aerobic glycolysis—the Warburg effect—in early breast cancer, which was proposed to provide a survival advantage whilst also supporting growth and invasion [194]. Importantly, it has been shown that the “Warburg phenotype” persists for many cell divisions even after the cells have returned to normal growing conditions, suggesting that the selected phenotypic state is rather stable and may have a profound effect on population dynamics during tumour progression [194]. Notably, it has been recently reported that exposing the mouse breast tumour cell line 4T1 to reduced oxygen levels leads to major changes in chromatin accessibility that may prompt the establishment of stable epigenetic states [195]. Strikingly, in this system it has been reported that chromatin accessibility profiles significantly vary depending on the duration of exposure to hypoxic conditions, suggesting that temporal dynamics also play a fundamental role in the establishment of distinct phenotypic states, which further expands tumour heterogeneity.

Importantly, besides modulating pathways that result in microenvironment remodelling and expand non-genetic heterogeneity, hypoxia may also lead to changes in the genetic background of a cancer cell population (Table 1). Indeed, it has been recently shown that hypoxia is associated with increased genomic instability in different types of tumours [166], suggesting that it may modify the genetic landscape of cancer cell populations, giving rise to new sub-clones that may further support its progression. Along those lines, in prostate cancer, hypoxia is characterized by higher rates of chromothripsis, mutations in PT53, the loss of PTEN and shorter telomeres [166], suggesting that besides metabolic non-genetic variability, hypoxia can lead to increased genetic intratumour heterogeneity. This, in turn, can lead to a hypoxia-mediated selection of more aggressive phenotypes that influence the tumour evolutionary path towards tumour growth, resulting in poor clinical outcomes [196]. Overall, the above-mentioned observations exemplify how a single environmental factor, in this case hypoxia, can lead to numerous complex paths that may support tumour progression through natural selection and adaptation.

Another striking example of niche construction in the context of cancer is the pre-metastatic niche formation (Table 1). During metastatic progression, cancer cells that invade new tissues must adapt to or modify a new environment in order to survive and thus colonize a new niche. Interestingly, it has been suggested that growth factors secreted by the primary tumour recruit bone marrow derived cell populations (marrow-derived hematopoietic progenitor cells) to distant pre-metastatic sites where these cells lead to changes in the local microenvironment that promote micro-metastatic lesion formation and determine organ-specific metastatic patterns [172,173]. These exciting data suggest that cancer cells may not only affect the microenvironment they actually reside in, but can also modify distant sites in a way that serves overall cancer progression.

Altogether, these examples clearly demonstrate the existence of a complex network of interconnections between cancer cells and their microenvironment. As discussed above, the microenvironment may support cancer cells in developing phenotypes that can, in turn, alter their microenvironment. Importantly, those alterations may evolve into functional adaptations that grant advantageous strategies for tumour progression, guiding cancer evolution.

### 5.5. Sub-Clonal Cooperation

As discussed in previous sections, tumours are characterized by extensive genetic and non-genetic heterogeneity, harbouring multiple distinct sub-clones. Intriguingly, it has been suggested that distinct malignant sub-clones present within a cancer cell population may not only compete but also undergo functional cooperativity via cell-to-cell contact, paracrine regulation and the modulation of the microenvironment [197], which induces tumour progression [198], promotes metastasis [180,181] and alters population dynamics in response to environmental cues [178] (Table 1). Using polyclonal breast cancer models, it has been shown that low-represented minor sub-populations expressing IL-11 (interleukin 11) and FIGF (Fos-induced growth factor) can drive tumour growth in other sub-clones and promote metastasis in a non-cell-autonomous manner apparently by modifying the local microenvironment [174,175]. Similar results were obtained using a bi-clonal breast cancer model containing genetically distinct luminal and basal sub-clones. This study has shown that the bi-clonal population was highly tumourigenic when transplanted into wild type mice, while monoclonal populations failed to cause tumours, suggesting a crucial role for sub-clonal cooperation in this particular context [176]. Furthermore, in a glioblastoma multiforme mouse model, a minor population that harbours mutant EGFR promoted the growth of EGFR wild type cells within the same tumour [177]. Interestingly, sub-clonal cooperativity has also been suggested to play an important role in the context of drug resistance. Along those lines, it has been demonstrated that colorectal cancer cells resistant to the EGFR blockade express TGF-α that sustains EGFR/ERK pathways and thus protects their sensitive counterparts from EGFR inhibitors [178]. These examples clearly demonstrate that distinct sub-clones residing within a single tumour can cooperate among themselves, promoting tumour progression while safeguarding intra-tumour heterogeneity that may be an essential pre-requisite of fast adaptations and cancer evolution as a whole.

Interestingly, by using several experimental models, including breast cancer, colon cancer and melanoma, Kim et al. have demonstrated that circulating tumour cells (CTCs) derived from metastatic lesions can colonize their tumours of origin in a process termed “self-seeding” [179]. Importantly, self-seeding cancer cells display more aggressive phenotypes compared to their descendants, and have been shown to promote tumour growth upon recruitment to the primary tumour. This effect was not associated with the outgrowth of the seeded cells, but was suggested to be a result of paracrine effects of the recruited sub-population on the overall population. These intriguing data challenge the unidirectional view of the metastatic process and suggest that phenotypically distinct metastasis-competent aggressive sub-clones can re-populate their site of origin, leading to an increased intra-tumour heterogeneity that supposedly shifts population dynamics towards more aggressive phenotypes through a cooperative network that fuels tumour progression.

Altogether, these examples challenge the cell-autonomous view of cancer progression, where only the most adapted “driver” phenotypes are selected; instead, distinct sub-clones may support each other’s survival upon facing variable conditions. This phenomenon might represent an adaptive strategy that enables the maintenance and stabilization of intra-tumour heterogeneity, which may be beneficial in case of future environmental changes.

### 5.6. Microbiome

Intriguingly, the microbiota that reside on the epithelial surfaces of our body also colonize tumours and have been suggested to have both systemic and local effects on cancer initiation, progression and response to drugs, adding another level of complexity to our understanding of the tumour microenvironment and its impact on cancer evolution (Table 1) [182]. In that regard, *Heliobacter pylori* is currently recognised by the World Health Organisation as a class I carcinogen that causes gastric cancer. Moreover, *Fusobacterrium spp* is associated with colorectal adenocarcinoma and colon cancer, while increased abundance of *Escherichia coli* has been observed in colon cancer patients [199,200,201]. These examples suggest a possible widespread role of the microbiome in cancer onset and progression. 

Interestingly, recent reports suggest that gut and intra-tumour microbiota might have an important role in modulating the efficacy of response to chemo- and immunotherapy [183,184,202]. Notably, by studying colon cancer models, Geller et al. have reported that intra-tumour bacteria—Gammaproteobacteria—can metabolize a chemotherapeutic agent, gemcitabine, into its inactive form, thereby granting tumour resistance [183]. Furthermore, the gut microbiome may have a crucial systemic effect in modulating the anti-cancer immune defence. Viaud et al. have shown that cyclophosphamide treatment induces the translocation of a defined set of Gram-positive bacteria species from the small intestine into secondary lymphoid organs, where it prompts the generation of “pathogenic” T helper 17 (pT_H_17) cells and memory T_H_1 immune mediated response. The strength of this immune response has been suggested to be associated with the anti-tumour efficacy of cyclophosphamide [184].

Furthermore, a recent study has provided compelling evidence highlighting the role of the microbiome in shaping the intra-tumour microenvironment and its crucial role in patient outcome. Along those lines, it was shown that the overall survival of pancreatic ductal adenocarcinoma (PDAC) patients correlates with the diversity of the tumour microbiome and the abundance of certain bacterial genera [185]. From the mechanistic perspective, it has been proposed that the intra-tumour microbiome may shape the immune microenvironment, leading to increased abundance of CD3+ and CD8+ T cells and granzyme B+, associated with immune activation in tumours of long term survivors (LTS) as compared to short term survivors (STS). Furthermore, faecal microbiota transplants (FMT) from LTS, STS and healthy donors have been shown to result in a striking difference in tumour growth in recipient mice. In that regard, mice receiving LTS-FMT displayed significantly reduced tumour growth in comparison to mice receiving STS-FMT. Further analysis revealed enrichment for CD8+ and activated T cells in the tumours of mice receiving LTS-FMT, while the tumours from STS-FMT mice were enriched in immunosuppressive regulatory T cells, suggesting that the microbiome might indeed have a profound local as well as systemic effect in shaping the tumour microenvironment and consequently modulating tumour progression. Interestingly, it has also been shown that a fungal community enriched in *Malassezia spp* residing within PDAC in humans and mice also contributes to tumour progression [203], highlighting that the microbiome is not limited to bacteria species and that fungi can also play a part in a complex network of functional interactions within the tumour microenvironment. Although more studies are required to find a causal relationship between specific microbiota species, cancer phenotypes and patient outcomes, increasing evidence suggests that the microbiome is an important and integral part of the tumour microenvironment that affects cancer onset, progression and survival in response to anti-tumour therapies, thus shaping tumour evolution.

Overall, cancer has long been viewed as a population of transformed cells that acquire a cell autonomous capacity for increased proliferation, motility, invasion and survival. However, an up-to-date wealth of evidence suggests that the cell-centred view of cancer is too simplistic, given the profound role of the complex multifactorial tumour microenvironment in cancer initiation, progression and metastasis. Therefore, the tumour microenvironment must be considered an essential dynamic and integral component of the “cancer tissue”, that through a complex multicellular network coevolves alongside the cancer cell population and shapes its evolutionary trajectory.

## 6. Therapeutic Avenues—Lessons from Cancer Evolution

Despite enormous efforts and significant advances in the development of anti-cancer therapeutic approaches ranging from radio- and chemotherapy to complex personalized treatments, most advanced cancers remain incurable. Notably, although most tumours initially respond well to a given treatment, they often develop resistant phenotypes, leading to relapse. In this light, most of the cancers display extensive intra- and inter-tumour heterogeneity and undergo dynamic evolution, which nowadays is believed to be a major contributor to resistant phenotypes and metastasis and, therefore, may underlie therapy failure. Furthermore, it is becoming increasingly evident that besides genetic heterogeneity, cancer cell populations display significant non-genetic heterogeneity that may lie at the basis of differential responses to various environmental cues and participate in the establishment of drug resistance and metastatic dissemination. Moreover, the complexity of the tumour microenvironment has a profound role in shielding tumours from drugs and the development of drug resistance, and may be a driver of phenotypic changes within cancer cell populations that have been linked to more aggressive cancer cell features.

However, even though the clinical relevance of ITH and its dynamic nature along the course of neoplastic disease are largely recognized, the therapeutic approaches accounting for dynamic tumour evolution are being developed at a very slow pace. As discussed in previous sections, this is mainly due the lack of adequate strategies that would allow a broad understanding of the extent of the genetic and non-genetic heterogeneity of an individual tumour and would enable the longitudinal tracing of phenotypic changes to monitor evolutionary changes throughout tumour progression in order to design adaptive therapeutic strategies. Importantly, such approaches may give a unique opportunity to target specific phenotypes at a particular stage of neoplastic disease, thus making an adaptive therapy the way forward for most cancers.

### 6.1. Targeting Genetic Heterogeneity

**Figure 5:**
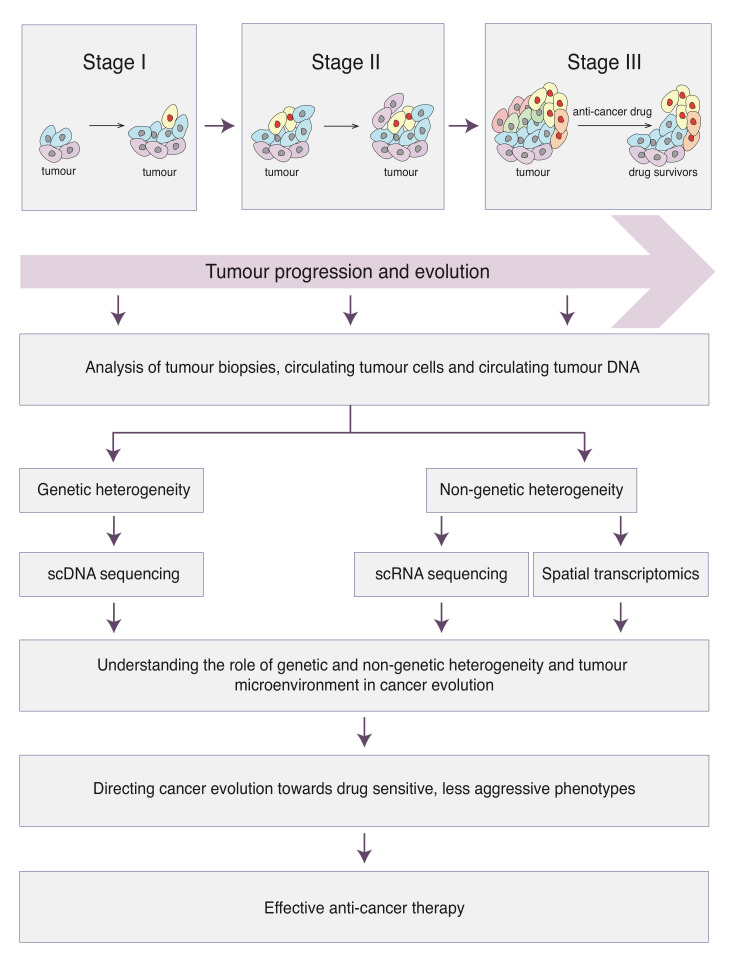
Novel systematic experimental approaches—powerful tools to diagnose cancer and monitor its progression. Tumours are rapidly-changing constantly-evolving open ecosystems. This feature of cancer development significantly affects the efficacy of anti-cancer therapy and patient outcomes. Therefore, it is crucial to monitor the dynamic changes taking place within a tumour along its development and upon drug treatment. Emerging experimental approaches that allow extraction and enrichment of circulating tumour cells and circulating tumour DNA may facilitate systematic analysis of tumour development at different stages of its progression. Furthermore, novel molecular biology technologies such as single-cell RNA and single-cell DNA sequencing as well as spatial transcriptomics provide an integral and more complete view on complex intra-tumour architecture and the dynamic changes taking place along cancer development. Overall, these may allow the design of more effective personalized therapeutic paradigms. The colour of the nuclei depicts a defined genotype; the colour of the cytoplasm shows distinct phenotypes that may result from genetic and non-genetic mechanisms.

Conventional single biopsy methods introduce significant sample bias and do not recapitulate the extent of spatial intra-tumour heterogeneity, potentially leading to an underestimation of the phenotypic variations coexisting within the tumour mass. Indeed, from the therapeutic point of view, this sole fact may underlie the reason why so many anti-cancer therapies fail. Furthermore, single biopsy tests do not provide any information regarding temporal changes that may accumulate as a result of natural selection and adaptation leading to the changes in population dynamics at a given time. To solve these issues, emerging non-invasive technologies that allow the serial sampling of circulating cancer cells and circulating cancer cell DNA from plasma represent appealing approaches that have the potential to overcome the above-mentioned limitations and could eventually be used to monitor evolutionary changes in individual tumours (Figure 5) [204,205,206,207,208,209,210]. These novel sampling strategies may enable the tracing of individual evolutionary trajectories by monitoring novel somatic mutations arising and/or being selected along the progression of a disease, throughout metastatic dissemination and upon therapy. Indeed, using a ctDNA sampling approach, it has been found that anti-EGFR therapy may lead to the acquisition of various somatic mutations in a number of genes, including various alterations in KRAS, NRAS, MET, EGFR, ERBB2, FLT3, and MAP2K1 genes that could cause the observed resistance to treatment [42,43,44]. These reports show that analysing ctDNA may indeed be a suitable strategy to monitor treatment-induced changes in ITH and adapt therapeutic approaches to specifically target emerging tumour-promoting phenotypes wherever possible.

On the other hand, despite the emergence of de-novo somatic mutations upon treatment, anti-cancer drugs may also select pre-existing drug refractory sub-clones [211], while eliminating sensitive ones. In that regard, although conventional cancer treatment strategies assume that the best therapeutic outcome is maximum reduction in tumour volume, changes in population dynamics due to the elimination of dominant sensitive sub-clones may result in a quick expansion of drug resistant and potentially more aggressive clones in an altered less competitive environment. This evolutionary phenomenon, known as “competitive release” [212], is at the basis of a proposed adaptive therapy where treatment aims to maintain a stable population of drug-sensitive cells, thus limiting the expansion of resistant clones [213]. In order to test this approach, preclinical breast cancer models were treated with variable doses of paclitaxel adjusted on the basis of tumour size (the dose was decreased if the volume reduced, and vice versa) for prolonged periods of time. Notably, such an approach kept the tumour volume at a constant level and supposedly enabled the maintenance of the persistent population of sensitive cells. This, in turn, could have allowed the population of sensitive cells to regrow when the treatment was discontinued, leading to reduced proliferation of the resistant sub-clones. These data suggest that applying evolution and ecology-based strategies to treat cancer as a chronic disease results in the maintenance of competitive interactions between drug-sensitive and drug-tolerant sub-clones and could significantly improve treatment efficacy [212,213].

### 6.2. Targeting Non-Genetic Heterogeneity

As discussed in the previous sections of this review, besides well-documented genetic heterogeneity, cancer cell populations are characterized by extensive non-genetic diversity. Although largely underestimated, mainly due to the lack of technological developments that would allow us to properly address its complexity and dynamic nature, evidence exists to argue that non-genetic heterogeneity may have a profound impact on tumour evolutionary history and thus on cancer patients’ outcome after therapy. Furthermore, the extent of non-genetic heterogeneity may go far beyond the complexity of genetic heterogeneity, given that a single genotype can produce a variety of phenotypes with drastically distinct behaviours in response to environmental cues, including anti-cancer drugs. Supporting this notion, it has recently been suggested that non-genetic heterogeneity is a major determinant of intra-tumour heterogeneity and evolutionary dynamics in lung cancer [25,31], highlighting the importance of this phenomenon in cancer biology.

Importantly, recently developed scRNA-seq sequencing technologies may help to unravel and further dissect intra- and inter-tumour non-genetic heterogeneity. For instance, by profiling 430 single cells from five primary glioblastomas, Patel et al. have shown that genetically identical cells (identified by quantifying copy number variations) display variable expression of several transcriptional signatures involved in oncogenic signalling, proliferation, stemness, hypoxia and immune response [214]. This example suggests that scRNA sequencing approaches could indeed be used to address the phenotypic heterogeneity of cancer cell populations and may be of crucial importance in designing adequate/personalized therapeutic approaches. Thus, we argue that scRNA profiling should be complementary to genotyping technologies as this will without a doubt expand the detection capacity of the complex diversity of phenotypic repertoire coexisting within individual tumours. Clearly, performing scRNA profiling using biopsies will face multiple limitations analogous to those specified above in regards to genotyping, such as a lack of spatial and temporal information concerning the dynamic nature of non-genetic heterogeneity. Thus, we believe that alternative approaches, such as serial scRNA profiling of circulating tumour cells, that could provide valuable insights into temporal dynamics and that would presumably mainly identify more aggressive metastatic and drug resistant phenotypes [37,215,216,217,218,219] should be considered as a complementary technique during therapy design.

As cancer cell populations are embedded within complex ecosystems, the spatial position and interactions with neighbouring cells may directly affect the cell state that determines its functional properties. Thus, carrying out transcriptomic profiling in dissociated tissue leads to the loss of spatial information and, as a result, restricts our capacity to understand complex intra-tumour organization, which may contain crucial information regarding functional relationships between phenotypically distinct sub-clones and the tumour microenvironment. Therefore, recently developed spatial transcriptomics is emerging as a powerful technology that provides quantitative, spatially-resolved (2D) transcriptomic profiling of individual tissue sections [220]. By positioning histological sections over millions of spatially barcoded capture oligonucleotides followed by scRNA sequencing [220], this strategy allows an unbiased visualization of cancer tissue architecture at the transcriptome level [220,221,222,223], that, at least in some cases, may have an important prognostic value [171,224,225,226]. Moreover, it may give a valuable opportunity to further investigate the complex crosstalk between different components of the tumour ecosystem to consequently evaluate its impact on cancer progression and evolution. Importantly, multiple reports suggest that natural selection in cancer cell populations may occur at the level of “total tissue performance”. Indeed, in colorectal cancer it has been demonstrated that a sub-clone resistant to the EGFR blockade expresses TGF-α, which, by sustaining EGFR/ERK pathways, protects their sensitive counterparts from EGFR inhibitors [178]. Similarly, using a zebrafish melanoma xenograft model, it has been demonstrated that in the presence of inherently invasive BRAF mutated (V600E) MITF^low^ WM266-4 cells, a poorly invasive sub-population of BRAF mutated (V600E) MITF^high^ 501mel cells co-invades alongside invasive cells, suggesting cooperative interactions between phenotypically distinct sub-populations [227]. These examples suggest that improved understanding of the intra-tumour spatial architecture may provide valuable insights into the evolutionary processes governed by mechanisms that maximize overall tissue performance, thus adding another facet to take into account when designing efficient therapeutic paradigms.

Furthermore, we propose that a detailed characterization of the extent and functional relevance of phenotypic heterogeneity generated by individual cancer related genotypes might allow light to be shed on the complex nature of phenotypic plasticity in the context of cancer. This knowledge can provide the molecular basis to further manage the establishment/propagation of distinct phenotypic states derived from a defined genotype, and potentially shift phenotypic variants towards drug sensitive and less aggressive phenotypes that may be targeted by existing therapeutic paradigms (Figure 4b). Thus, the further development/refinement of multimodal high-throughput technologies that would allow the simultaneous analysis of the genetic and phenotypic status is of utmost importance in order to develop strategies accounting for both genetic and non-genetic heterogeneity.

### 6.3. Targeting the Tumour Microenvironment

Given the importance of the tumour microenvironment in shaping tumour phenotypes and its evolutionary trajectory, multiple strategies are being developed to eradicate tumours by means of altering their microenvironment. As discussed in the previous sections of this review, very often the tumour microenvironment is characterized by drug refractory properties due to multiple mechanisms, including its poor perfusion and vascularization, that limit the efficacy of drug delivery and create a gradient of drug diffusion. Along those lines, Olive et al. have shown that the combinatorial treatment of PDAC tumours with a Hedgehog pathway inhibitor (IPI-926) and gemcitabine led to an increased intra-tumoural vascular density, resulting in an elevated intra-tumoural concentration of gemcitabine and leading to a transient stabilization of the disease [228]. Thus, modifying the intra-tumour vascular network may have a significant impact on the efficacy of drug delivery. Furthermore, it may reduce hypoxia, which is associated with poor prognosis through a number of well-characterized mechanisms, thus improving therapeutic outcome.

On the other hand, stromal components of the tumour microenvironment have been shown to have an important role in modifying cancer cell phenotypes and affecting the efficacy of several anti-cancer therapeutic paradigms [229]. For instance, it has been recently reported that the immune subtypes of triple negative breast cancer determine its sensitivity to immunotherapy [171], thus the identification of those subtypes may be of great importance in designing effective therapeutic strategies. Notably, the application of scRNA sequencing approaches to identify immune subtypes within tumours is emerging as a powerful and reliable tool that may be used to help adequate therapeutic design for individual patients. Interestingly, novel strategies that may allow the modification of the stromal components of the tumour microenvironment may help to circumvent resistance to anti-cancer therapy. For instance, in a targeted antibody-based anti-cancer approach tested in a humanized model of B-cell leukaemia, it was shown that leukaemic cells infiltrate the bone marrow, rewire the tumour microenvironment and inhibit the macrophage-mediated killing of antibody-tagged cancer cells, thus resulting in low efficacy of an antibody-based therapeutic approach [230]. However, a combinatorial treatment of cyclophosphamide (CTX) and antibodies has been shown to circumvent this phenomenon and significantly increase the efficacy of the antibody treatment. In that regard, it has been suggested that CTX induces acute secretory activating phenotype (ASAP), producing CCL4, IL8, VEGF, and TNF-α from treated cancer cells, resulting in drastic changes in the tumour microenvironment. In this context, in turn, ASAP induces macrophage infiltration and increases phagocytic activity in the bone marrow, which prompts a more effective antibody response. This example shows that the release of stress-related cytokines triggered by a chemotherapeutic agent may change the nature of the bone marrow from a treatment-refractory to a treatment-sensitive niche, and thus has the potential to change the evolutionary trajectory of a given cancer type.

Taken together, these data show that the tumour microenvironment, which often supports tumour progression, can be turned into a hostile—anti-cancer—environment, enabling effective tumour eradication. Thus, targeting the tumour microenvironment in combination with other therapeutic approaches may represent a strategy to direct cancer evolution towards drug susceptible phenotypes—a path that, indeed, must be explored.

## 7. Conclusions

Tumours are constantly evolving open ecosystems. Upon cancer initiation, newly arising cancer cell populations undergo clonal expansion followed by genetic and non-genetic phenotypic diversification, which is a substrate for natural selection in a complex dynamic and multifactorial environment of a given tumour niche. Due to the fact that cancer cell populations, in addition to their habitats, are not static but rapidly changing coevolving systems, tumours display highly variable patterns of sub-clonal architecture resulting in extremely complex ecological systems, which represents a major obstacle in effective cancer eradication. Therefore, when dealing with such a complex system that is barely understood, the development of efficient therapeutic approaches may seem hopeless. However, rapid technological progress in developing novel molecular biology techniques, detection methods and analytical tools provides an opportunity to perform multifactorial analysis of individual tumours, which is of crucial importance to investigate the complexity of tumour tissue and the functional role of its individual components that will undoubtedly aid the development of personalized therapeutic approaches. Furthermore, though we are only now beginning to understand cancer biology from the evolutionary point of view, we believe that further in-depth studies of cancer development from the ecological and phylogenetic perspective may enable us to develop a theoretical evolutionary framework, which might allow the prediction and potential direction of the evolutionary path of cancer cell populations and, consequently, allow us to be one step ahead in our fight against cancer.

## Figures and Tables

**Table 1:** Role of tumour microenvironment in cancer evolution.

Component of the Tumour Microenvironment	Example	Impact on Cancer Biology	References
	Increased stiffness of ECM	Promotes translocation of EMT-regulating transcription factors into the nucleus and drives EMT in breast cancer and PDAC.	[160,161]
	PLX4720 mediated ECM remodelling	Triggers increased integrin β1/FAK/Scr signalling in BRAF-mutated melanoma cells. This is followed by ERK signalling activation that results in the establishment of resistance to PLX4720.	[162]
**ECM and other non-cellular components**	Vemurafenib mediated fibronectin deposition	Results in increased AKT/PI3K activation, which abrogates the cytotoxic response to the BRAF inhibitor.	[163]
	Hypoxia	Activates gene expression programs that facilitate cancer cell survival, induce invasive growth, reduce immune responses and promote vascularization in hypoxic regions. Hypoxia is associated with increased genomic instability in different type of tumours.	[164,165] [166]
	Inflammatory environment	May promote tumour growth directly by inducing cancer cell proliferation, or indirectly by down-modulating the immune response, activating tumour-promoting innate immunity signalling, impairing the induction of angiogenesis and removing constrains in tissue remodelling. Induces the expression of tumour promoting factors.	[167] [168,169]
	Monocytes and macrophages	Production of TNF-α by a macrophage population triggers MITF expression, resulting in cancer cell resistance to MAPK-inhibitors.	[170]
**Immune cells and other cellular components**	Macrophages	Macrophage-enriched subtype of triple negative breast cancer displays sensitivity to immunotherapy.	[171]
	Neutrophils	Neutrophil-enriched subtype of triple negative breast cancer shows resistance to immunotherapy.	[171]
	Cancer associated fibroblasts	Extensive deposition of extra cellular matrix that causes desmoplasia.	[158]
	Marrow-derived hematopoietic progenitor cells	Upon recruitment to distant pre-metastatic, sites these cells modify the local microenvironment to promote micrometastatic lesions.	[172,173]
	Pro-metastatic cooperation	In polyclonal breast cancer models, low-represented subpopulations expressing IL-11 (interleukin 11) and FIGF (Fos-induced growth factor) can drive proliferation in other sub-clones and promote metastasis.	[174,175]
	Tumorigenic cooperation	A bi-clonal breast cancer model containing genetically distinct luminal and basal sub-clones is highly tumourigenic when transplanted into wild type mice, while monoclonal populations fail to cause tumours.	[176]
**Sub-clonal cooperation**	Growth promoting cooperation	In a glioblastoma multiforme mouse model, a minor population that harbours mutant EGFR can promote growth of EGFR wild-type cells within the same tumour.	[177]
	Drug resistance	Colorectal cancer cells resistant to EGFR blockade express TGF-α that sustains EGFR/ERK pathways and thus protects their sensitive counterparts from EGFR inhibitors.	[178]
	“Self-seeding”	Circulating tumour cells derived from metastatic sites can colonize their tumours of origin and promote tumour growth.	[179]
	Cancer initiation and progression	*Heliobacter pylori* can cause gastric cancer. *Fusobacterrium spp* is associated with colorectal adenocarcinoma and colon cancer. Increased abundance of *Escherichia coli* is observed in colon cancer patients. *Malassezia spp* residing within PDAC contributes to tumour progression.	[180,181,182]
	Drug resistance	Intra-tumour bacteria—Gammaproteobacteria—can metabolize a chemotherapeutic agent, gemcitabine, into its inactive form, thereby granting tumour resistance in colon cancer models.	[183]
**Microbiome**	Modulation of cancer immune response	Upon cyclophosphamide treatment, a defined set of Gram-positive bacteria species translocates from the small intestine into secondary lymphoid organs where it promotes the generation of “pathogenic” T helper 17 (pTH17) cells and memory TH1 immune mediated response.	[184]
	Shaping the intra-tumour microenvironment	The specific microbiome of PDAC patients may increase the abundance of CD3+ and CD8+ T cells and granzyme B+, which correlates with immune activation in tumours of long-term survivors (LTS) as compared to short-term survivors (STS).	[185]

## Appendix A

### Appendix A.1. Allele Specific Gene Expression in Cancer Initiation and Progression

A variable cell specific gene expression program that leads to the establishment of metastable phenotypes may result from allele-specific gene expression mechanisms, such as dose compensation, imprinting and/or monoallelic gene expression. Allele-specific variability arises when two alleles within the same cell are differentially regulated, leading to persistent differences in allele function. Notably, to the best of our current knowledge, genomic imprinting is strikingly relevant in the context of cancer.

Genomic imprinting is a non-genetically controlled mechanism of gene expression regulation that causes allele-specific (maternal or paternal) expression of a number of genes [231,232]. In mammals, monoallelic expression ensures precise dosage control over the protein levels generated from imprinted genes that encode for factors involved in embryonic/placental growth and adult metabolism. The regulation of imprinted genes mainly relies on the DNA methylation status of certain chromosomal regions—Imprinting Control Regions (ICRs)—which is established during gametogenesis [231]. The methylation status of ICRs is stable and heritable upon cell divisions in somatic cells ensuring precise and consistent control of gene expression. From the mechanistic perspective CCCTC-binding factor (CTCF) plays an important role in the regulation of gene expression from imprinted regions [233]. In that regard, CTCF acts as a molecular insulator and through binding to non-methylated ICRs prevents distant enhancers from accessing promoters enabling allele specific expression. Furthermore, CTCF has been suggested to play a crucial role in preventing de-novo methylation of differentially methylated regions (DMRs) thus exerting control over large scale expression programs [231,233].

Notably, the loss of imprinting (LOI) through either loss or gain of DNA methylation leads to mis-regulation of imprinted genes (activation of normally silenced alleles, or silencing of normally transcribed alleles) and results in a number of developmental abnormalities [231]. Moreover, LOI has been suggested to be the most abundant alteration in different types of cancer, including chronic myeloid leukaemia, ovarian tumours, Wilms’s tumours, colorectal cancer, Barrett’s oesophagus carcinoma, oesophageal cancer, lung adenocarcinoma, meningioma, osteosarcoma, hepatoblastoma, glioma, Ewing’s sarcoma, laryngeal squamous cell carcinoma and rhabdomyosarcoma [234,235].

Perhaps the most common and well-characterized LOI associated with a wide range of cancer types is the LOI at IGF2/H19 locus (70% on Wilm’s tumours, 30% of colon cancer). This event leads to the activation of a normally silent allele of a growth-promoting gene—IGF2 (insulin growth factor 2)—followed by the subsequent expression of both IGF2 alleles. The imprinted IGF2/H19 locus expresses both IGFR2 from a paternal allele and H19 non-coding RNA with known growth suppressive properties from a maternal allele. Importantly, LOI leads to the biallelic expression of IGF2 that correlates with altered methylation levels of the IGF2/H19 locus. Along those lines, it has been suggested that hypermethylation of the H19 allele enables the recruitment of CTCF to a proximal ICR followed by the activation of IGF2 expression from the silenced allele while concomitantly inhibiting H19 transcription. An alternative model suggests that aberrant methylation of the regions upstream of IGF2 plays a crucial role in LOI-mediated IGF2 expression [236,237,238]. Consequently, these changes lead to the overexpression of IGF2 and the down regulation of H19, which may give cells a growth and fitness advantage over their normal counterparts and therefore promote cancer progression.

Notably, LOI associated with cancer is not limited to the IGF2/H19 locus [233]. Nearly 10% of Wilm’s tumours display LOI at the gene encoding for the cyclin dependent kinase inhibitor 1C (CDKN1C) involved in G1/S-phase arrest [239,240]. Furthermore, LOI at the RAS related gene—DIRAS3 has been reported for breast and ovarian cancer [241], whist LOI at the mesoderm specific transcript homologue gene (MEST) have been found in breast, lung and colon cancer [242]. A recent study analysing the stability of ICRs has reported that the majority of imprinted domains are highly unstable in human cancers [233] highlighting that the loss of imprinting which disrupts fine tuning of gene dosage may represent a widespread non-genetically controlled phenomenon that supports neoplastic disease in different contexts.

Interestingly, several studies performed on Wilm’s tumours, ovarian and colorectal cancer reported an increased frequency of LOI of the IGF2 encoding gene in the tissue surrounding the tumour mass suggesting that LOI may be an early tumorigenic event. Along those lines it has been proposed that LOI of IGF2 may initially occur in a sub-population of normal cells and might represent a “pre-existing” alteration that predisposes cells to oncogenic transformation. In those lines, up-regulation of IGF2 expression may lead to an increased expansion of a sub-population that had undergone LOI, which in turn increases the potential acquisition of somatic mutations and may result in cancer initiation. The latter hypothesis goes in line with an epigenetic progenitor model of cancer onset [243] in which the first step of cancer initiation is proposed to be an epigenetic change/event. In this model, abnormal epigenetic events take place in stem/progenitor cells within a given tissue in response to cell intrinsic or cell extrinsic processes. In that context, it is believed that the accumulation of the aforementioned epigenetic abnormalities is further followed by one or several oncogenic mutations that may result in cancer initiation. Thus, these observations allow hypothesising that the loss of imprinting in susceptible cell sub-populations may promote an initial branching towards a malignant phenotype by providing them with a growth advantage and increasing the chances of further cancer-promoting alterations to occur either at the genetic or epigenetic level.

Interestingly, from the mechanistic perspective allele-specific gene expression is often associated with specific chromatin reorganization [97]. For example, imprinted immunoglobulin genes are sequestered at the nuclear periphery, whilst their transcribed counterparts are located nearer the centre of the nucleus [244]. In another example, hundreds of inactive olfactory receptor genes are phase-separated into a single heterochromatin structure, while their enhancers are organized into a super-enhancer to ensure monoallelic expression distinct to each cell [105,106]. Finally, as higher order chromatin structures have also been associated with imprinted domains, and given the dynamic and variable nature of the genome organization, it is only logical to suggest that this mechanism might have a widespread nature in enabling differential gene expression programs that may contribute to the establishment of metastable phenotypic states and intra-tumour non-genetic heterogeneity.

### Appendix A.2. Molecular Basis of EMT and MET

EMT is induced by specific paracrine signals such as the transforming growth factor (TGF-β), WNT ligands, epidermal growth factor (EGF), interleukin (IL-6), fibroblast growth factor (FGF), and hepatocyte growth factor (HGF), often secreted by the tumour stromal microenvironment that activate several signalling pathways (TGF-β, NOTCH, Wnt, ERK, p38MAPK, PI3K-AKT, JNK). The aforementioned signalling pathways converge to activate the EMT program mainly by inducing the expression of EMT-TFs (e.g SLUG, SNAIL, ZEB1, ZEB2, TWIST1, TWIST2) or by regulating microRNAs (e.g miR-34, miR-200) and long non-coding RNAs (lncRNA) directly involved in EMT control [123]. Molecularly, the repression of E-cadherin (encoded by the *CDH1* gene) is considered a hallmark of the EMT program, thus its expression is highly regulated by multiple mechanisms involving transcription factors, histone/DNA modifications and post-transcriptional regulation of the main EMT components. Altogether, the concert of all these pathways enables the stepwise transition and generation of metastable and stable states during EMT [122,123]. Along those lines, several computational models have been developed to explore the dynamic nature of phenotypic switches orchestrating the EMT program [245,246,247,248] Importantly, it has been predicted that EMT is a sequential two step multi-stable program, governed by tightly balanced double-negative SNAIL1/miR-34, ZEB/miR-200 feedback loops that function as bi-stable switches (hysteresis) [245]. Briefly, initial TGF- β stimulation leads to the accumulation of *snail1* mRNA, however, an increase in protein level takes place later when miR-34 mediated translational repression is abrogated. This leads to the partial inhibition of E-cadherin and partial activation of N-cadherin and consequently results in the establishment of an intermediate quasi-epithelial/mesenchymal reversible state. An increase in TGF-β stimulation leads to further induction of SNAIL1 expression that in turn triggers *zeb1* expression. Similarly to SNAIL, the expression of ZEB protein is delayed due to miR-200 mediated translational repression. Eventually, when both proteins SNAIL1 and ZEB are expressed, a full inhibition of E-cadherin and a full activation of N-cadherin takes place resulting in the establishment of a stable mesenchymal state [245]. Notably, according to another model ZEB/miR-200 acts as a tri-stable circuit and represents a key regulatory loop in EMT induction, while a monostable SNAIL/miR-34 module functions as a noise buffering integrator [246]. Importantly, increasing the complexity of the modelling by adding more regulatory elements results in more intermediate states appearing between the epithelial and mesenchymal states [249,250]. This goes in line with recent experimental data suggesting that EMT is a transition through multiple intermediate hybrid states that are characterized by different chromatin landscapes and gene expression profiles that display variable cellular plasticity and increased invasiveness and metastatic potential [127]. Along those lines, computational models also predict that the heterogeneity in the EMT transition dynamics is a result of extrinsic and intrinsic noise [245,251]. Furthermore, it has been proposed that the acquisition and maintenance of the epithelial to mesenchymal transition in a population of cancer cells can result from the noise in partitioning of EMT determinants during cell division [251], which might be lying at the basis of multiple intermediate states coexisting between the epithelial and mesenchymal phenotypes. Importantly, the predictions of the aforementioned models have been experimentally validated in several cancer types, though it has been observed that not all cellular systems display the same EMT dynamics [129,247,249,252], a phenomenon that may have a crucial implication in cancer progression and metastasis [247]. Overall, multidisciplinary computational modelling as well as experimental reports demonstrated that EMT is a dynamic multistep process that consists of metastable and stable phenotypic states.

Notably, from the mechanistic perspective, it has been suggested that chromatin re-arrangements may play an essential role in the establishment and maintenance of those states. Along those lines, it has been proposed that the upregulated in response to external stimuli, sequence specific EMT-TFs, are initially recruited to the promoters of the genes participating in EMT, which in turn primes the recruitment of chromatin-modifying machinery that further establish EMT gene expression programs. In that regard, it has been found that in pancreatic and colon adenocarcinoma an EMT-TF, SNAIL, interacts with EZH2 and SUZ12 (polycomb complex 2 members) and associates with the CDH1 promoter, which in turn leads to the trimethylation of H3K27 (H3K27me3) resulting in CDH1 silencing. This in turn may lead to the recruitment of chromodomain-containing proteins including CBX2, CBX4, CBX8 and BMI1 (polycomb complex 1 members), which may further repress target genes [253].

The role of histone acetyltransferases and deacetylases in the EMT program has also been evaluated. In particular, it has been shown that in a pancreatic cancer model, the histone deacetylases HDAC1 and HDAC2 that catalyse the removal of acetyl groups from histone 3 lysine residues (9 and 14) and thus promote gene silencing, are recruited by SNAIL to the promoter of the CDH1 gene [254]. Importantly, different repressive histone modifications have been suggested to be associated with less or more stable silencing. For instance, H3K27me3 is known to cause silencing that can be easily converted into an active state in response to specific differentiation stimuli, whereas H4K9me3 is associated with constitutive heterochromatin, leading to more stable silencing. Moreover, some evidence suggests the existence of bivalent promoters that might be characterized by the coexistence of repressive H3K27me3 and active H3K4me3 modifications within the same promoter [255,256,257]. Interestingly, it has been shown that cells residing in a “more” epithelial state and express E-cadherin bear H3K4me3 modification at the CDH1 promoter, whereas cells transitioned into a “more” mesenchymal phenotype with attenuated E-cadherin expression bear both H3K27me3 and H3K4me3 modifications, that presumably permits a fine-tuning and dynamic regulation of the EMT program [255]. Notably, the existence of bivalent promoters must be taken with caution, given the possibility that the observed coexistence of distinct marks may reflect differential representation of those marks within different alleles as well as within different cells.

Current models aiming to summarize the mechanisms orchestrating the epithelial-to-mesenchymal transition suggests that, during the EMT program, cells undergo a gradual stepwise change associated with the generation of metastable and stable phenotypic states [122]. In that context, chromatin modifications have been hypothesised to be a major contributor in supporting the dynamic nature of this program. In that regard, it has been proposed that upon EMT-inducing stimuli, epithelial genes are initially repressed by gaining H3K27me3 resulting in a bivalent configuration with H3K4me3. Although the repressive modification is gained, this state is believed to be highly plastic and easily reversible, which may further be turned into an intermediate, and rather stable, mesenchymal state associated to the loss of H3K4me3. The latter state may be followed by the acquisition of a more stable repressive modification—H3K9me3—associated with constitutive heterochromatin, thus anchoring the repressive state. Furthermore, persistent EMT-inducing signals may result in H3K9me3 mediated recruitment of DNMTs leading to DNA methylation, creating a highly stable silencing of epithelial genes/traits that can be propagated over many cell generations. Overall, this model may explain the dynamic functional adaptation that tumour cells endure throughout the invasion-metastasis cascade. In that regard, the metastable quasi-epithelial and quasi-mesenchymal states might govern cell detachment and invasion into the surrounding tissues, whereas the rather stable mesenchymal phenotype, that can perhaps be sustained in the absence of constitutive EMT-inducing signals, may enable intravasation, migration, extravasation and further invasion into a new tissue and could grant the initial survival signals upon residing within a new niche.

Interestingly, recent study has revealed the potential mechanism that enables reversion of the mesenchymal phenotype by triggering MET. It has been found that compounds, which activate adenylate cyclase (cholera toxin and forskolin) result in increased levels of the second messenger—adenosine–3′5′–monophosphate (cAMP) that activates protein kinase A (PKA), which in turn activates its substrate—the demethylase PHF2. This leads to a reprograming cascade that promotes demethylation and derepression of epithelial genes resulting in a phenotypic switch and acquisition of epithelial traits [127]. Notably, understanding the mechanisms that underlie MET is of the utmost relevance, as it may open new therapeutic avenues to explore. For instance, it could be envisaged to target the cells that exhibit tumour-initiating and metastatic properties to force their reprograming towards an epithelial phenotype. This therapeutic approach could result in a reduced risk of tumour dissemination and could in turn make a population of cancer cells more susceptible to conventional therapeutic paradigms.
